# Diastereoselective Amplification of a Mechanically Chiral [2]Catenane

**DOI:** 10.1021/jacs.1c06557

**Published:** 2021-07-29

**Authors:** Kenji Caprice, Dávid Pál, Céline Besnard, Bartomeu Galmés, Antonio Frontera, Fabien B. L. Cougnon

**Affiliations:** † Department of Organic Chemistry, University of Geneva, 30 Quai Ernest-Ansermet, 1211 Geneva, Switzerland; ‡ Laboratory of Crystallography, University of Geneva, 24 Quai Ernest-Ansermet, 1211 Geneva, Switzerland; § Department de Química, Universitat de les Illes Balears, Carretera de Valldemossa km 7.5, 07122 Palma de Mallorca, Baleares, Spain

## Abstract

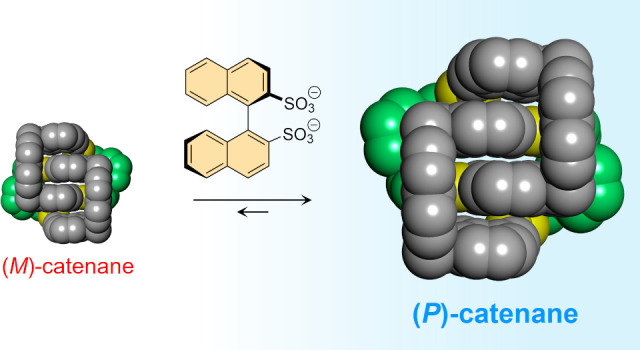

Achiral [2]catenanes composed of rings with inequivalent sides may adopt chiral co-conformations. Their stereochemistry depends on the relative orientation of the interlocked rings and can be controlled by sterics or an external stimulus (e.g., a chemical stimulus). Herein, we have exploited this stereodynamic property to amplify a mechanically chiral (*P*)-catenane upon binding to (*R*)-1,1′-binaphthyl 2,2′-disulfonate, with a diastereomeric excess of 85%. The chirality of the [2]catenane was ascertained in the solid state by single crystal X-ray diffraction and in solution by NMR and CD spectroscopies. This study establishes a robust basis for the development of a new synthetic approach to access enantioenriched mechanically chiral [2]catenanes.

The enantioselective synthesis of chiral mechanically interlocked molecules (MIMs)^1−4^ has made spectacular progress in recent years, enabling the development of sophisticated molecular machines with functional applications in stereoselective catalysis,^5^ sensing,^6^ and chiroptical switching.^7^ These applications rely on the ability of MIMs to express their chirality in different ways when the relative position of the interlocked components changes, an unusual property that is not accessible with traditional covalent systems. Despite major synthetic achievements, the production of chiral MIMs generally remains tedious and requires elaborate multistep syntheses, involving the independent synthesis of several low symmetry components.^3,4^ New strategies that can provide access to complex chiral molecular machines in a simple, cost-effective manner are therefore needed.

A potential way to address the above-mentioned limitations lies in the work of Puddephatt et al.^8^ and Marinetti, Sauvage, and co-workers^9^ who have shown that combining two rings with inequivalent faces produces a pair of axially chiral enantiomers (Figure 1a). These enantiomers are configurationally stable and cannot interconvert without breaking a covalent bond. Their stereochemistry is comparable to that of axially chiral allenes, with one notable difference: the axial chirality of the [2]catenane arises exclusively from the presence of the mechanical bond connecting the rings, a phenomenon referred to as *mechanically axial chirality*.^10^ This observation implies that chiral MIMs can be produced by combining components that are identical, achiral, and nondirectional. Each of these features evidently reduces the synthetic cost of the final compound. However, examples of such [2]catenanes are scarce. In addition, the control of mechanically axial chirality is highly challenging and has never been achieved: to date, only racemates have been obtained.

**Figure 1:**
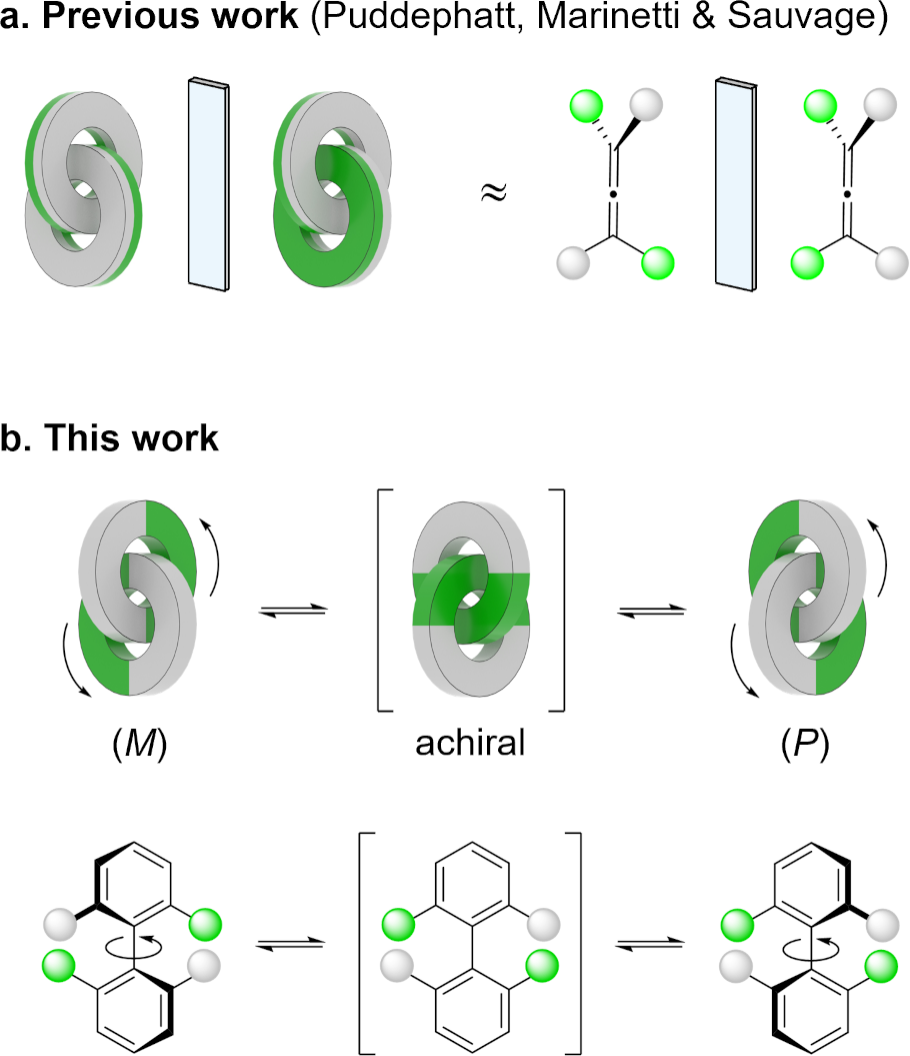
Two situations in which mechanically axial chirality may be encountered. (a) The stereochemistry of [2]catenanes composed of rings with inequivalent faces (previous work) is similar to that of axially chiral allenes. (b) The stereochemistry of [2]catenanes composed of rings with inequivalent sides (contribution of this study) is closer to atropisomerism.^11^

We now present a simple alternative approach to access enantioenriched mechanically axially chiral [2]catenanes. Figure 1b shows a [2]catenane composed of rings with inequivalent sides, rather than inequivalent faces. Such [2]catenanes are commonly found in the literature.^12,13^ They are generally considered to be achiral because they possess a mirror plane when the mirror planes of the individual rings are brought to coincide. Yet, moving the rings on either side of the mirror plane generates axially chiral co-conformations.^14,15^ These co-conformations may be either left-handed (*M*) or right-handed (*P*), depending on the relative orientation of the rings. In contrast with the situation presented in Figure 1a, the motion of the rings now results in the interconversion of the enantiomeric co-conformers.^16,17^ This behavior is somewhat reminiscent of atropisomerism.^11^ If the motion of the rings is unconstrained, which is typically the case in previous reports, the individual co-conformers interconvert too rapidly to be detected and the [2]catenane displays no sign of chirality. Here we show that the chiral co-conformers can become observable when the motion of the rings is hindered. More importantly, we show that the dynamic nature of this system can be exploited to easily amplify a single enantiomer in response to a chemical stimulus.^18^

A sterically hindered [2]catenane composed of rings with inequivalent sides was assembled following a dynamic combinatorial approach (Figure 2).^19^ In water, amphiphilic building blocks frequently self-assemble into catenanes to minimize the overall hydrophobic surface area in contact with the environment.^13,20^ Quinolinium-based dialdehyde **1** (1 mM) and dihydrazide **2** (1 mM) were solubilized in water at pH 5. The solution was stirred overnight at 70 °C, allowing for the reversible formation of acylhydrazone linkages between the building blocks. On the following day, HPLC analysis disclosed the near-quantitative conversion of the starting materials into [2]catenane **3**, which was isolated by semipreparative HPLC as a trifluoroacetate salt (**3**·4CF_3_CO_2_) in 83% yield.

**Figure 2:**
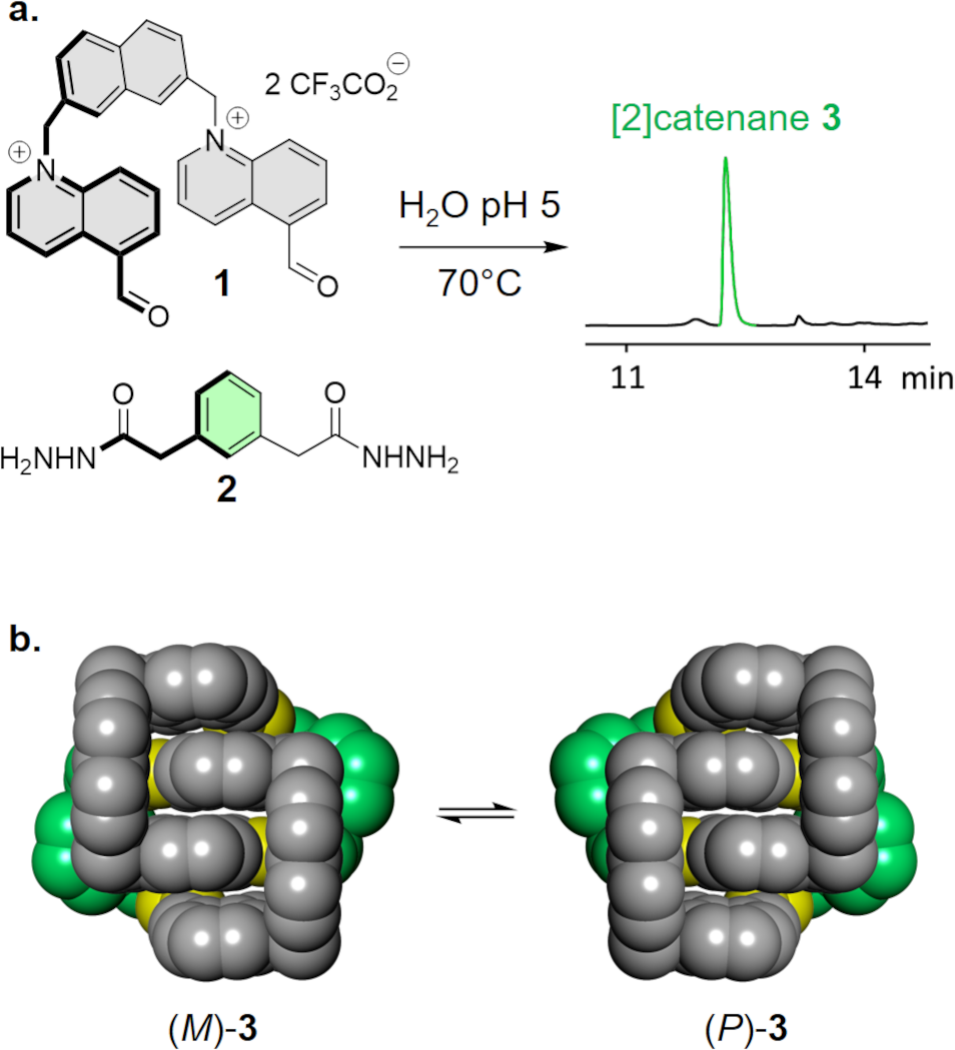
(a) Synthesis of [2]catenane **3** from dialdehyde **1** (in gray) and dihydrazide **2** (in green). The HPLC chromatogram shows the purity of the crude mixture at the end of the reaction. (b) Crystal structure of the enantiomers (*M*)-**3** and (*P*)-**3**. The acylhydrazone linkages are colored in yellow. Hydrogens are omitted for clarity.

Tandem mass spectrometry (Figure S4)^21^ rapidly confirmed that [2]catenane **3** was composed of two identical macrocycles, each resulting from the condensation of one dialdehyde **1** (in gray) and one dihydrazide **2** (in green).

Attempts to obtain single crystals of **3**·4CF_3_CO_2_ from aqueous solutions were unsuccessful. Fortunately, slow vapor diffusion of isopropyl ether in a concentrated acetonitrile solution of the hexafluorophosphate salt **3**·4PF_6_ (prepared by following an anion exchange protocol described in the SI), yielded single crystals suitable for X-ray diffraction. [2]Catenane **3** is a particularly compact structure. The optimum packing of the aromatic units results in a decrease of solvent accessible surface area of ca. 37% compared to that of two non-interlocked macrocycles, explaining the high yield of the [2]catenane assembly. The cavity of the individual rings is narrow, oblong, and delimited by large aromatic walls. Steric demands impose considerable constraint on the relative orientation of the rings, which can only be interlocked if the quinoliniums stack as depicted in Figure 2b. The [2]catenane is thus locked into a well-expressed axially chiral state. Of all the possible co-conformers, only the enantiomers (*M*)-**3** and (*P*)-**3** are present and alternate in the three dimensions of the crystal lattice (Figure 3).

**Figure 3:**
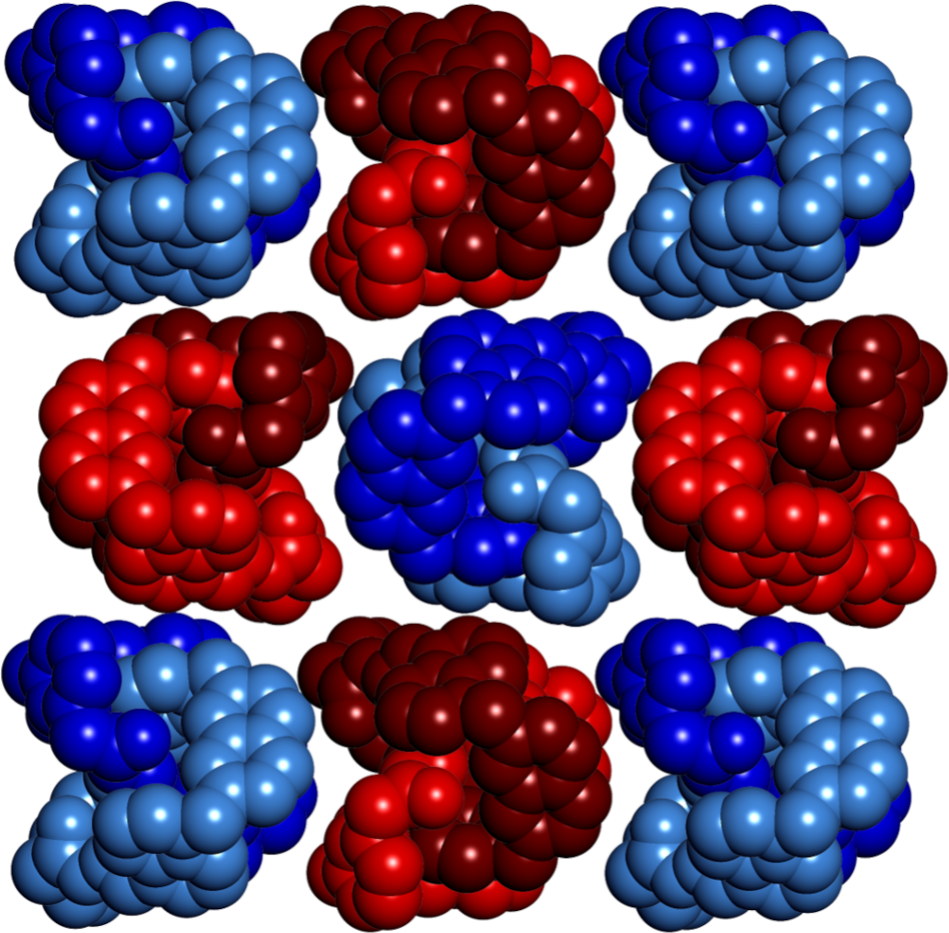
Crystal packing showing the alternation between (*M*)-**3** (in red) and (*P*)-**3** (in blue). Hydrogens and counterions are omitted for clarity.

The ^1^H NMR spectrum of **3**·4CF_3_CO_2_ in D_2_O (Figure 4a) comprises sharp, well-dispersed resonances, and it does not significantly change between 278 and 338 K (Figure S5). These features confirm that [2]catenane **3** has little conformational freedom. The spectrum is consistent with the *C*_2_-symmetrical structure observed in the solid state. The two rings are equivalent, and all the protons of an individual ring are inequivalent.

**Figure 4:**
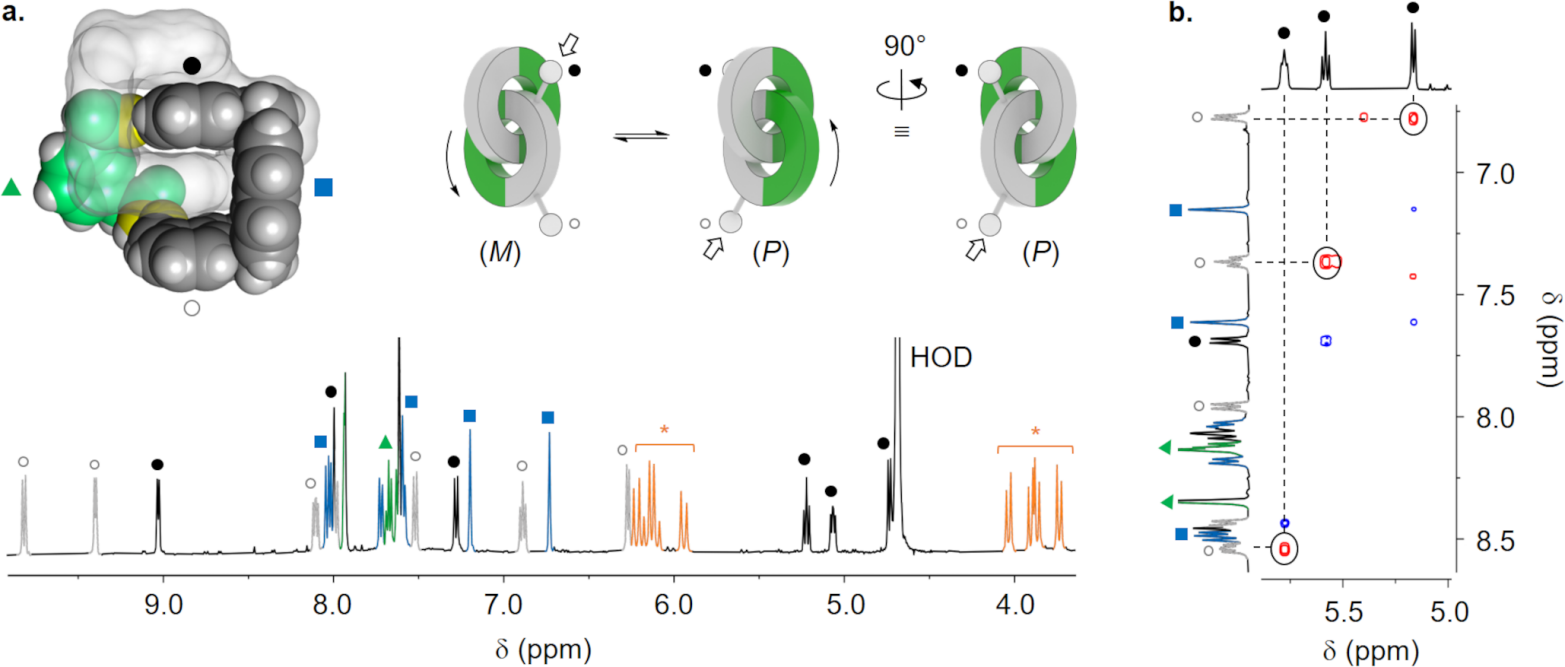
(a) ^1^H NMR spectrum (D_2_O, 500 MHz, 298 K) of [2]catenane **3** highlighting the signals corresponding to the inner quinolinium (●), outer quinolinium (○), naphthalene (■) and phenylene (▲) protons. Diastereotopic methylene protons are labeled with a star (∗). The cartoon representations show that the enantiomerization results in an exchange between inequivalent quinolinium protons (● ↔ ○). (b) Corresponding exchange cross-peaks are observable in the NOESY spectrum (338 K, d_8_ = 300 ms). The full interpretation of the NMR spectra can be found in the SI.

The protons of the inner quinoliniums, buried in the stack, are substantially upfield-shifted compared to those of the outer quinoliniums. Moreover, the methylene protons are diastereotopic. In conclusion, the [2]catenane also exists as a racemic mixture of (*M*)-**3** and (*P*)-**3** in solution. The spectrum shows no evidence of any other co-conformers.

The enantiomers (*M*)-**3** and (*P*)-**3** may interconvert through either mechanism of ring pirouetting or ring circumrotation.^22^ In any case, the enantiomerization results in the exchange of the inner and outer quinoliniums (Figure 4a). Their inequivalence implies that the process is slow on the NMR time scale. Nevertheless, the presence of exchange cross-peaks between pairs of inequivalent quinolinium protons in the 2D NOESY spectrum (Figure 4b) allowed for the determination of the enantiomerization rate constants between 313 and 338 K.^23^ The Eyring plot generated the enthalpy (Δ*H*^⧧^ = +61 kJ·mol^–1^) and entropy (Δ*S*^⧧^ = −83 J·mol^–1^·K^–1^) of activation and the energy barrier (Δ*G*^⧧^_298 K_ = +85 kJ·mol^–1^).

The barrier to interconversion is high enough to enable the NMR resolution of the (*M*)- and (*P*)-enantiomers. Indeed, addition of 0.07 equiv of potassium disulfonate^24,25^ (*R*)-**4** to the racemic solution of **3**·4CF_3_CO_2_ (1.13 mM, D_2_O/CD_3_CN 1:1)^26^ resulted in the separation of each signal of the [2]catenane into two signals at δ_*M*_ and δ_*P*_ (Figures 5a and S16).

**Figure 5:**
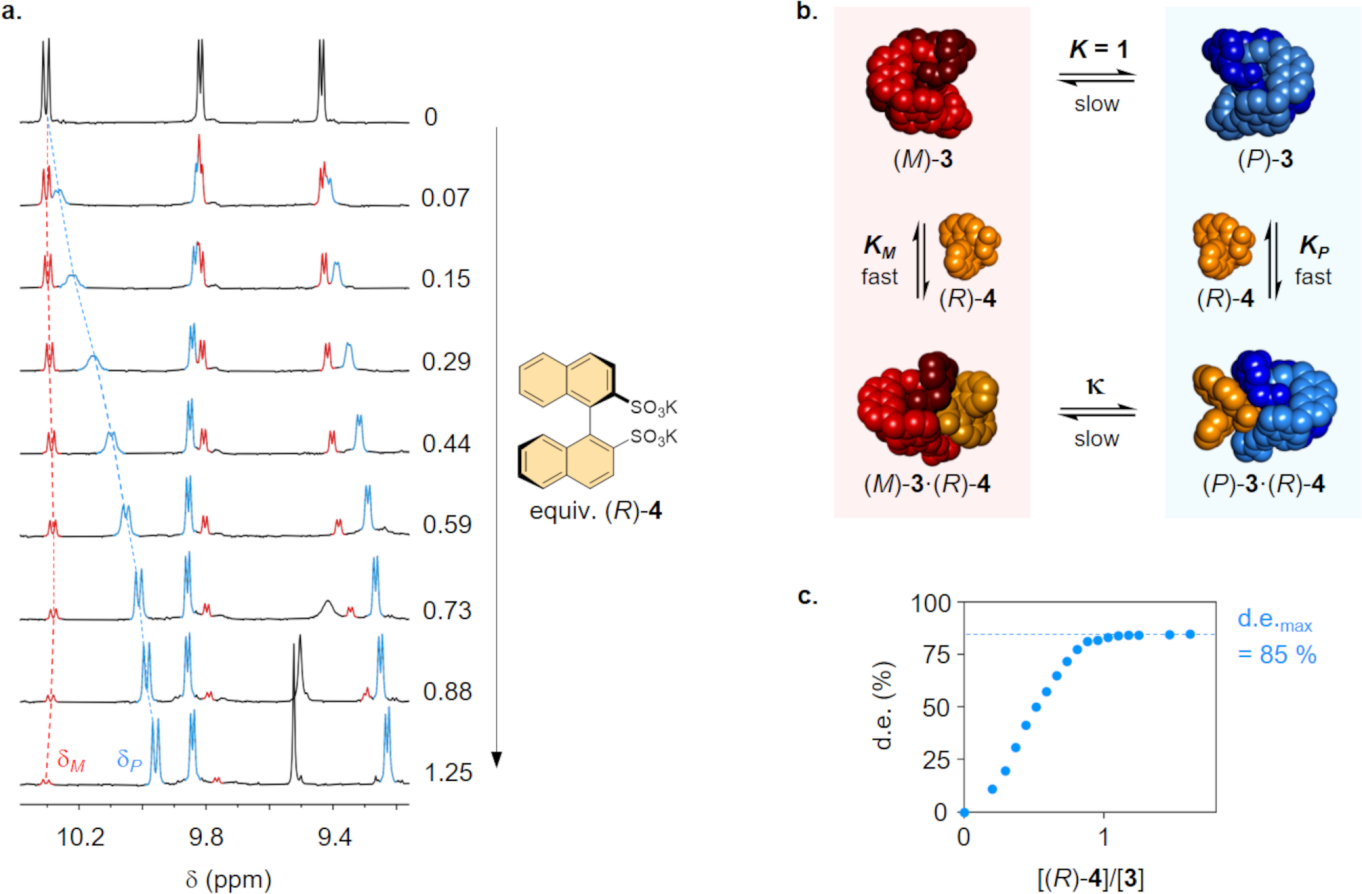
Amplification of the diastereomeric complex (*P*)-**3**·(*R*)-**4**. (a) ^1^H NMR titration of (*R*)-**4** to a solution of [2]catenane **3** (1.13 mM, D_2_O/CD_3_CN 1:1, 500 MHz, 298 K). (b) Representation of the equilibria involved in the diastereoselective amplification. (c) Evolution of the diastereomeric excess in the course of the titration.

As the quantity of disulfonate (*R*)-**4** added increased, the signals at δ_*M*_ and δ_*P*_ further separated and their relative intensity noticeably changed. This phenomenon indicates that (*R*)-**4** preferentially binds one of the two enantiomers and shifts the equilibrium toward the formation of the most stable diastereomeric complex. The integration of the separated signals provided a direct measurement of the diastereomeric excess, which increased with [(*R*)-**4**]/[**3**] until it reached a maximum value, de_max_ = 85% (Figure 5c).

DFT calculations revealed that the amplified diastereomeric complex was (*P*)-**3**·(*R*)-**4** (Figure 6). The [2]catenane possesses a binding site where (*R*)-**4** can nest and form four short and directional hydrogen bonds with the acylhydrazone NHs. Disulfonate (*R*)-**4** binds both enantiomers, but fits better in the binding site of (*P*)-**3** than in that of (*M*)-**3** (Figure S24). Since (*P*)-**3**·(*R*)-**4** is virtually the only species observable in the spectrum at the end of the titration, it was fully characterized by ^1^H and ^13^C NMR (Figures S17–S20).

**Figure 6:**
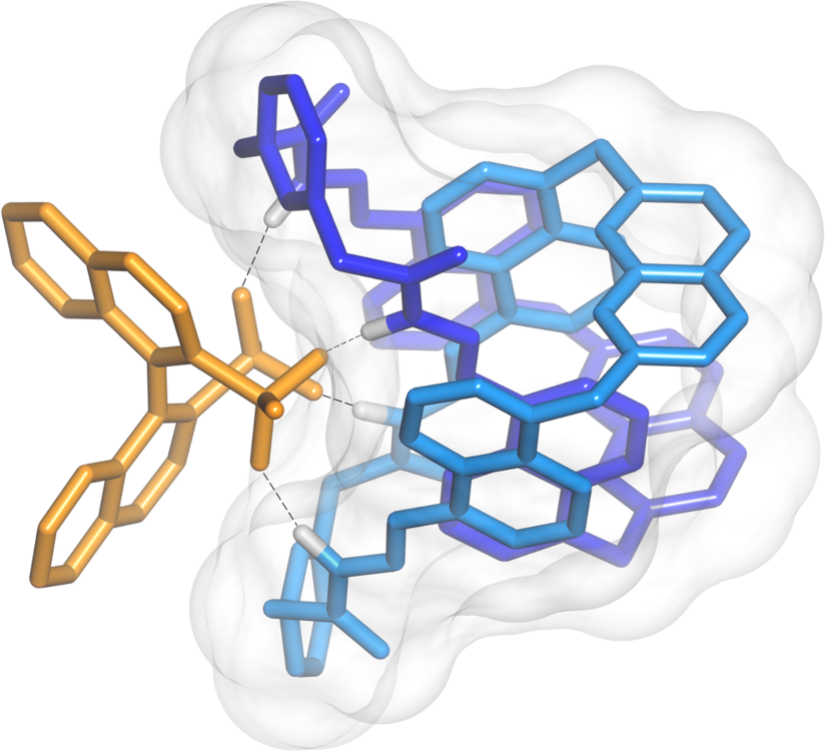
BP86-D3/def2-TZVP optimized geometry of the diastereomeric complex (*P*)-**3**·(*R*)-**4**.

If (*M*)-**3** and (*P*)-**3** bind (*R*)-**4** with association constants *K*_*M*_ and *K*_*P*_, respectively (Figure 5b), the diastereomeric excess at saturation is determined by the ratio κ = *K*_*P*_/*K*_*M*_, which represents the selectivity of the anion for one of the two co-conformational states. The value κ = 12.3 calculated from de_max_ indicates that *K*_*P*_ is an order of magnitude superior to *K*_*M*_ and corresponds to a difference of stability Δ*G*_298K_^°^ = 6.2 kJ·mol^–1^ between the two diastereomeric complexes. In principle, both *K*_M_ and *K*_P_ can be obtained by fitting the plot de = *f*([(*R*)-**4**]/[**3**]). The derivatization of the corresponding equations is detailed in the SI. However, the early saturation of the titration curve prevented the accurate determination of the individual association constants. It was only possible to conclude from this first experiment that *K*_*M*_ and *K*_*P*_ were greater than 10^4^ M^–1^.

The diastereoselective amplification was confirmed by circular dichroism (Figure 7). As expected, the racemic [2]catenane **3**·4CF_3_CO_2_ (38 μM) exhibited no CD signal in H_2_O/CH_3_CN 1:1. Upon addition of disulfonate (*R*)-**4**, an induced CD (ICD) signal appeared, consisting of a strong negative Cotton effect at 303 nm and a weaker negative Cotton effect at 364 nm. The intensity of the ICD increased with the number of equivalents of (*R*)-**4** until it reached a maximum at [(*R*)-**4**]/[**3**] ≈ 10. This maximum intensity corresponds to the diastereomeric excess de_max_ = 85% previously measured by NMR, as this value is independent of the range of concentration at which the titration is performed. Taking this information into account, the ICD intensity could be converted into a diastereomeric excess at any stage of the titration (Figure 7, inset). This time, fitting the titration curve successfully afforded the association constants *K*_*P*_ = 2.6 × 10^5^ M^–1^ and *K*_*M*_ = 2.1 × 10^4^ M^–1^.

**Figure 7:**
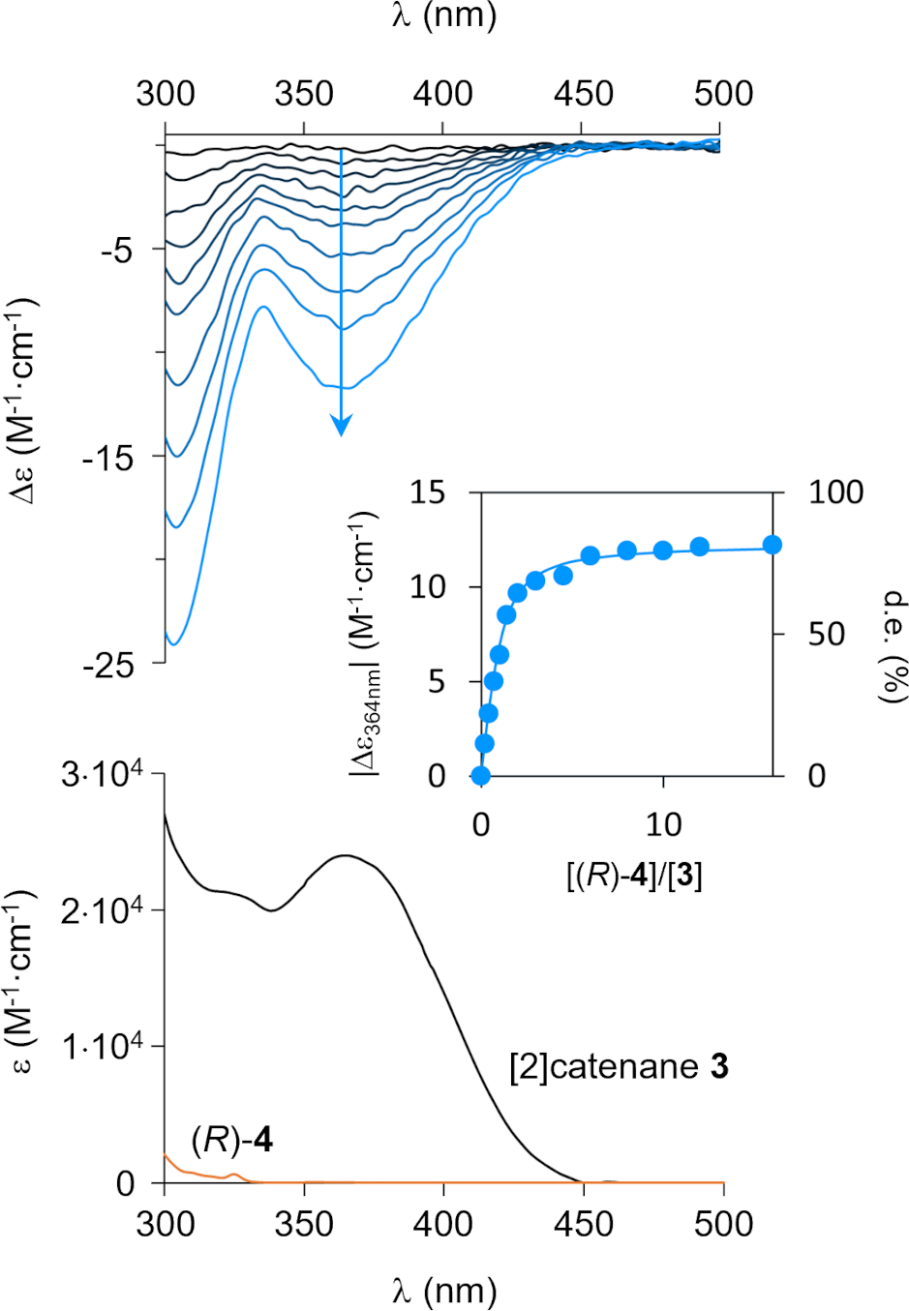
(bottom) UV–visible spectra of [2]catenane **3** and (*R*)-**4** (H_2_O/CH_3_CN 1:1). (top) ICD spectra of **3** (38 μM, H_2_O/CH_3_CN 1:1) in the presence of 0–15 equiv of (*R*)-**4**. Inset: ICD amplitude at 364 nm as a function of the number of equivalents of (*R*)-**4**.

In conclusion, we have described a simple approach to access mechanically chiral [2]catenanes in enantioenriched form. This approach relies on the ability of [2]catenanes composed of rings with inequivalent sides to adopt achiral and chiral co-conformations in dynamic exchange. If the relative orientation of the rings can be controlled by an external stimulus, it is possible to reversibly switch the [2]catenane between achiral, (*M*), and (*P*) states.^18^ Here we have exploited this property to bias the population of co-conformers in favor of a (*P*)-catenane. The stereodynamic nature of this system is its most distinctive feature. However, we anticipate that increasing the steric bulk of the rings will slow down the enantiomerization rate enough to enable the isolation of the enantioenriched [2]catenane in a configurationally stable form. These results demonstrate a promising route to the construction of new chiral molecular devices for advanced applications in catalysis, molecular recognition, and material sciences.

## Supporting Information Available

Experimental details, spectroscopic and X-ray crystallography data of the [2]catenane, computational methods. and Cartesian coordinates of the optimized diastereomeric complexes (PDF)

## Accession Codes

CCDC 2091887 contains the supplementary crystallographic data for this paper. These data can be obtained free of charge via www.ccdc.cam.ac.uk/data_request/cif, or by emailing data_request@ccdc.cam.ac.uk, or by contacting The Cambridge Crystallographic Data Centre, 12 Union Road, Cambridge CB2 1EZ, UK; fax: +44 1223 336033.

## Abbreviations

NMR: nuclear magnetic resonance
CD: circular dichroism
HPLC: high performance liquid chromatography
