# Supplementary material for: Diastereoselective Amplification of a Mechanically Chiral [2]Catenane
Source: J Am Chem Soc. 2021 Jul 29;143(31):11957–62. doi: 10.1021/jacs.1c06557 (PMC8397304; doi:10.1021/jacs.1c06557)
Supplement: Supplementary file 1 — ja1c06557_si_001.pdf [file ja1c06557_si_001.pdf]

Supporting Information for:

Diastereoselective Amplification of a Mechanically Chiral [2]Catenane

Kenji Caprice,[a] Dávid Pál,[a] Céline Besnard,[b] Bartomeu Galmés,[c] Antonio Frontera,[c] and Fabien B. L. Cougnon[a]†*

[a] Department of Organic Chemistry, University of Geneva, 30 Quai Ernest-Ansermet 1211 Geneva 4, Switzerland

[b] Laboratory of Crystallography, University of Geneva, 30 Quai Ernest-Ansermet 1211 Geneva 4, Switzerland

[c] Department de Química, Universitat de les Illes Balears, Carretera de Valldemossa km 7.5, 07122 Palma de Mallorca, Baleares, Spain

Corresponding Author
* fabien.cougnon@nuigalway.ie

Present Address
† School of Chemistry, National University of Ireland, University Road, Galway, H91 TK33, Ireland.

Table of Contents

1. Methods S3

2. Synthesis and characterization of the building blocks S5

3. Characterization of [2]catenane **3** S8

4. Determination of the thermodynamic parameters of enantiomerization of [2]catenane **3** by Exchange Spectroscopy (EXSY) S19

5. Diastereoselective amplification and characterization of the diastereomeric complex (*P*)-**3**·(*R*)-**4** S21

6. Crystallography S29

7. DFT calculations S31

8. References S38

1. Methods

All reagents and solvents were purchased from commercial sources and were used without further purification. Reagents for synthesis and titrations were purchased from Alfa Aesar (quinoline-5-carboxaldehyde, trifluoroacetic acid, silver trifluoroacetate, ammonium hexafluorophosphate), Sigma–Aldrich [2,7-bis(bromomethyl)naphthalene, 1,3-phenylenediacetic acid, hydrazine monohydrate], and Tokyo Chemical Industry Co [dipotassium (*R*)-1,1'-binaphthyl-2,2'-disulfonate].

Synthesis of [2]catenane 3. Building blocks **1** (1 mM) and **2** (1 mM) were dissolved in water (10 mL, 0.01% trifluoroacetic acid). The solution was heated at 70 ºC overnight and the resulting [2]catenane was isolated by semi-preparative HPLC. Each fraction was frozen as soon as it was collected and lyophilized. The [2]catenane **3** was obtained as a fluffy yellow powder (**3^{4+}·4$CF_3CO_2^-$**, isolated yield: 83%).

HPLC analyses. Chromatograms were obtained from a reverse-phase XBridge (150 x 4.6 cm, 3.5 µm particle size) connected to an HPLC Agilent 1260 (injection volume: 1 µL; flow rate: 1 mL/min at room temperature; mobile phase: linear gradient 5–60% CH_3CN in H_2O with 0.1% trifluoroacetic acid over 30 min; absorbance monitored at 254 nm; data processing: HP ChemStation).

Semi-preparative HPLC. The same HPLC Agilent 1260 was used for semi-preparative isolation, with a reverse-phase XBridge (150 x 10 cm, 5 µm particle size). Injection volume: 100 µL to 900 µL. Flow rate of 3 mL/min at room temperature. Absorbance was monitored at 254 nm. Data processing: HP ChemStation.

Anion exchange. The purified [2]catenane (**3^{4+}·4$CF_3CO_2^-$**) was solubilized in the minimum amount of milli-Q water and a saturated aqueous solution of NH_4PF_6 was added dropwise until no further precipitation occurred. The precipitate (**3^{4+}·4PF_6^-**) was isolated by centrifugation, washed several times with milli-Q water and dried in the lyophilizer.

NMR analyses. Spectra were measured using a Bruker Avance III 500 MHz spectrometer equipped with a 5-mm DCH ^{13}C-^{1}H/D helium-cooled cryogenic probe. All signals were internally referenced to the solvent residue. NMR solvents (D_2O, CD_3CN, and DMSO-d_6) were purchased from Cambridge Isotope Laboratories and ARMAR Isotopes.

Exchange Spectroscopy EXSY. The thermodynamic parameters of enantiomerization of [2]catenane **3** were obtained by EXSY. The data were collected using a mixing time of 300 ms, at which exchange cross-peaks were observed, and compared to reference spectra with a minimal mixing time of 3 ms, at which exchange cross-peaks were not observed. The rate constant k_1 was calculated from the intensity of cross- and diagonal-peaks, using the software EXSYCalc developed by Mestrelab research. [S1] The exchange cross- and diagonal-peaks

e↔e′ were chosen for the quantitative determination of these parameters because they were isolated and could be easily integrated.

MS analyses. MS analyses were performed by the mass spectrometry core facility of the University of Geneva on a QSTAR Pulsar (Qq TOF, ionisation mode: ESI (+)).

UV and CD analyses. UV analyses were performed on a Jasco V-650 Spectrophotometer and CD spectra was recorded on a Jasco J-815 CD Spectrometer.

2. Synthesis and characterization of the building blocks

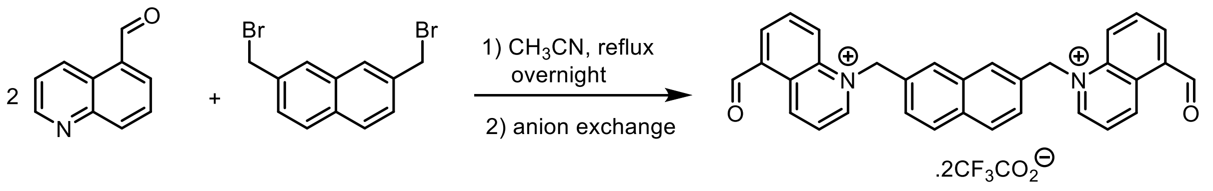

Synthesis of building block 1. Quinoline-5-carboxaldehyde (100 mg, 0.64 mmol) and 2,7-bis(bromomethyl)naphthalene (100 mg, 0.32 mmol) were solubilized in acetonitrile (5 ml) and refluxed overnight. The precipitate was collected by filtration, washed with small volumes of acetonitrile (10 mL total) and dried under vacuum. The product was then dissolved in water with 1 equiv. of silver trifluoroacetate and stirred overnight in aluminium foil. The resulting suspension was centrifuged to remove any trace of finely suspended AgBr. The aqueous phase was lyophilized, yielding $\mathbf{1^{2+}{\cdot}2CF_3CO_2^-}$ as a beige powder. Yield: 142 mg, 64%. m. p.: 240 ºC (decomposition). HR-MS (ESI+) calculated for $C_{32}H_{24}N_2O_2^{2+}$ $[M]^{2+}$ (*m/z*) 234.0919, found 234.0922. ^{1}H NMR (DMSO-d_6, 500 MHz, 298 K) δ (ppm): 10.53 (s, 2H), 10.24 (d, *J* = 8.8 Hz, 2H), 9.86 (d, *J* = 6.0 Hz, 2H), 8.79 (d, *J* = 9.1 Hz, 2H), 8.58 (d, *J* = 7.2 Hz, 2H), 8.48 (dd, *J* = 8.8, 6.0 Hz, 2H), 8.36 (dd, *J* = 9.1, 7.2 Hz, 2H), 7.98 (d, *J* = 8.5 Hz, 2H), 7.78 (d, *J* = 1.8 Hz, 2H), 7.57 (dd, *J* = 8.5, 1.8 Hz, 2H), 6.58 (s, 4H). ^{13}C NMR (DMSO-d_6, 125 MHz, 298 K) δ (ppm): 193.0, 151.1, 144.4, 137.8, 137.0, 134.9, 132.4, 132.4, 132.4, 132.3, 128.9, 127.4, 126.4, 125.7, 124.9, 124.8, 60.8.

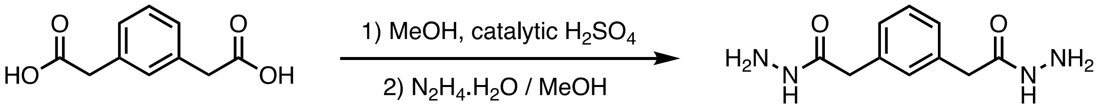

Synthesis of building block 2. 1,3-Phenylenediacetic acid (100 mg, 0.515 mmol) was dissolved in methanol (5 mL). A catalytic amount of sulfuric acid was added (20 μL) and the mixture was refluxed overnight to obtain the corresponding methyl diester. At the end of the reaction, all the volatiles were removed. The diester was directly resolubilized in methanol (3 mL) and hydrazine monohydrate (3 mL) and the reaction mixture was heated at 70 ºC for 4 h. All the volatiles were removed and the product was dried in vacuum. Yield: 112 mg, 98%. m. p.: 193–194 ºC. HR-MS (ESI+) calculated for $C_{10}H_{15}N_4O_2^+$ $[M]^+$ (*m/z*) 223.1195, found 223.1199. ^{1}H NMR (DMSO-d_6, 500 MHz, 298 K) δ (ppm): 9.20 (s, 2H, NH), 7.17–7.23 (m, 1H), 7.07–7.15 (m, 3H), 4.23 (br s, 4H, NH_2), 3.30 (s, 4H). ^{13}C NMR (DMSO-d_6, 125 MHz, 298 K) δ (ppm): 169.4, 136.1, 129.5, 128.0, 127.1, 40.4.

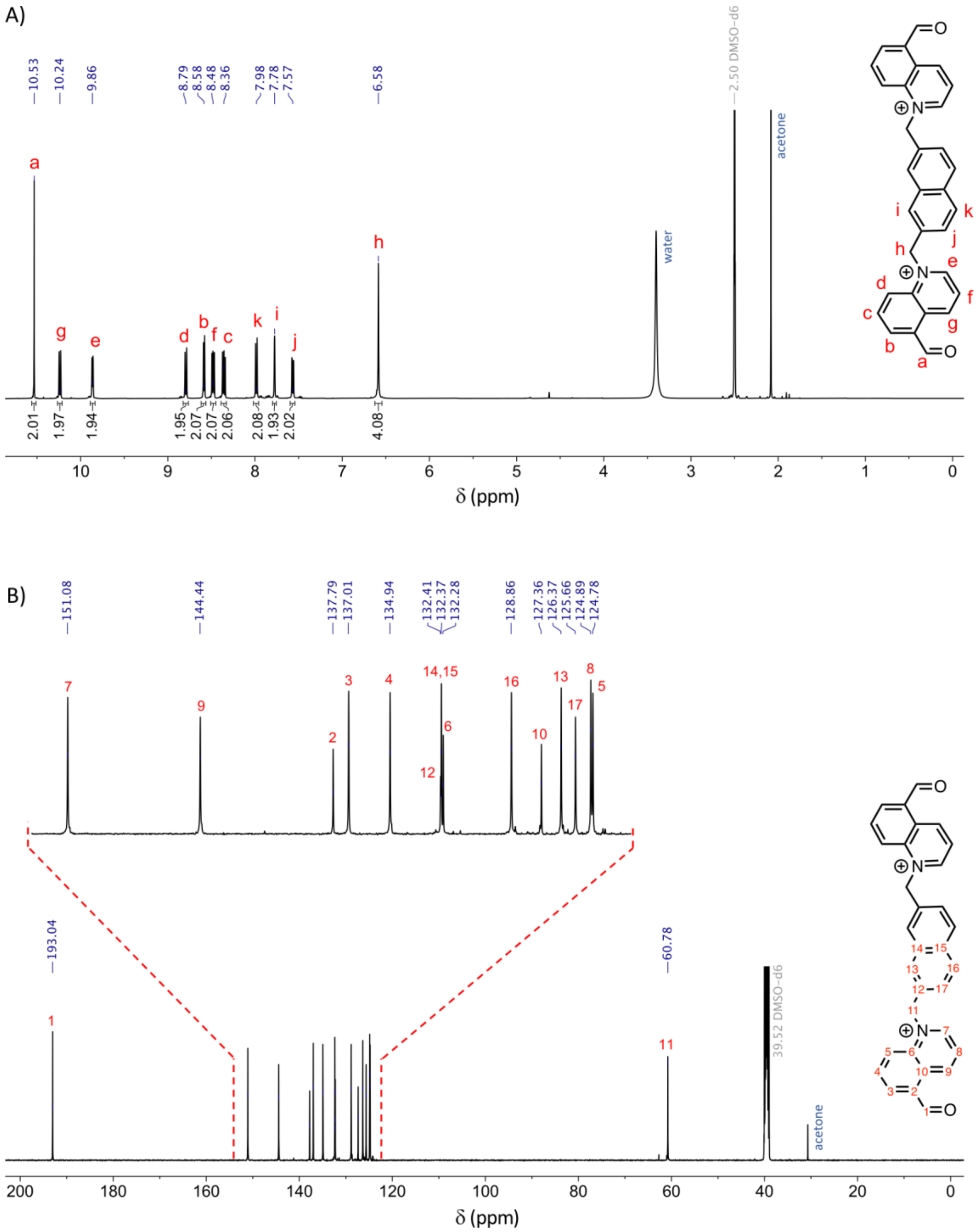

Figure S1. **A)** ^{1}H NMR (DMSO-d_6, 500 MHz, 298 K) and **B)** ^{13}C NMR (DMSO-d_6, 125 MHz, 298 K) spectra of building block **1**.

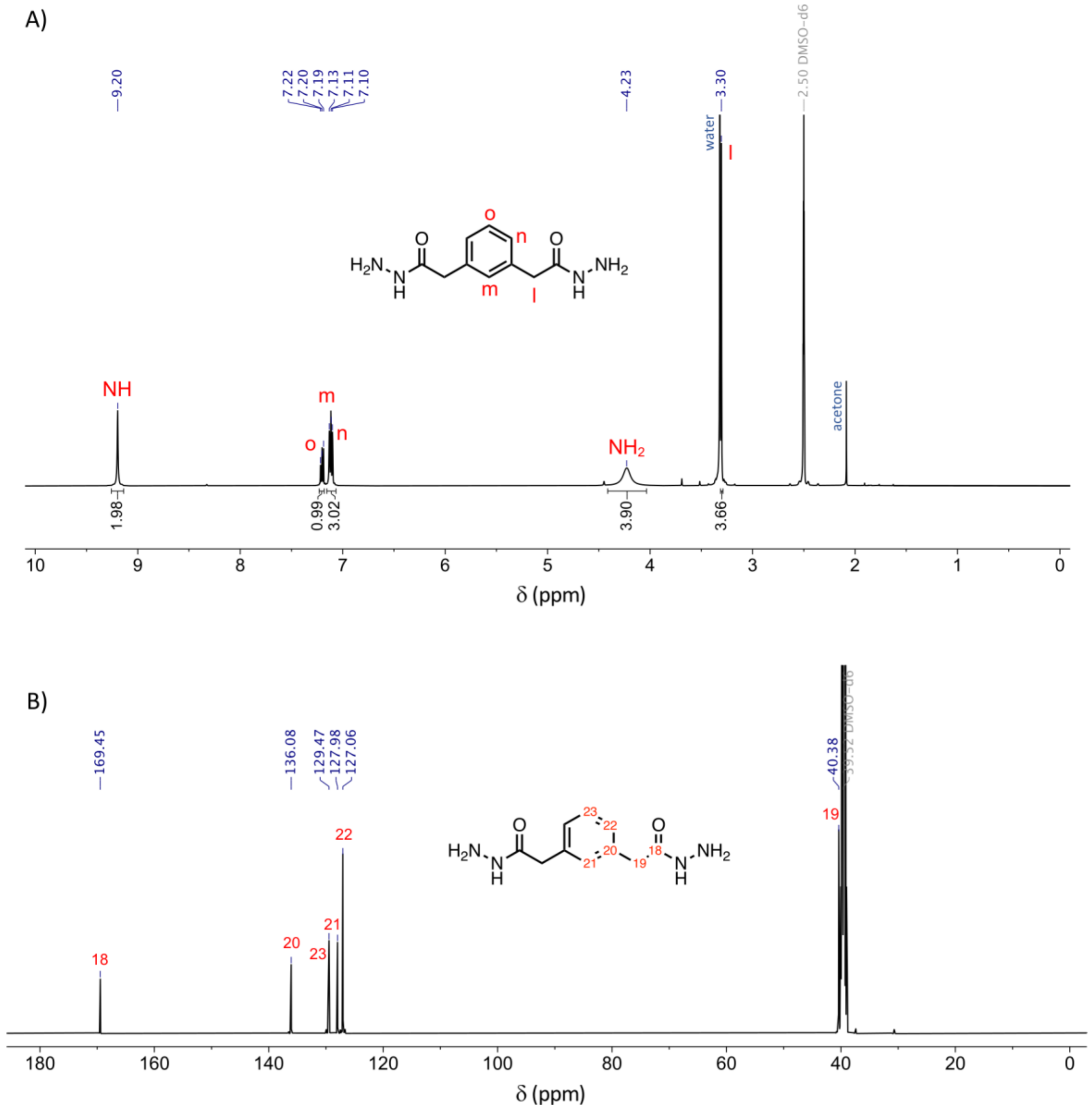

Figure S2. **A)** ^{1}H NMR (DMSO-d_6, 500 MHz, 298 K) and **B)** ^{13}C NMR (DMSO-d_6, 125 MHz, 298 K) spectra of building block **2**.

3. Characterization of [2]catenane 3

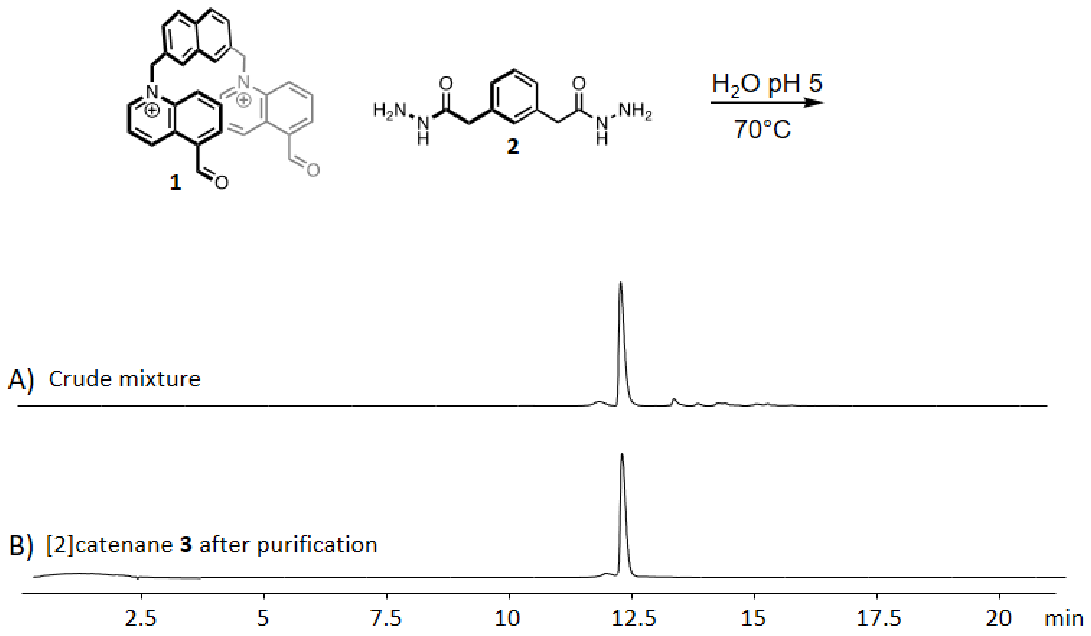

Figure S3. Reverse-phase HPLC traces of **A)** the crude mixture generated from building blocks **1** and **2** before purification and **B)** the isolated [2]catenane **3**.

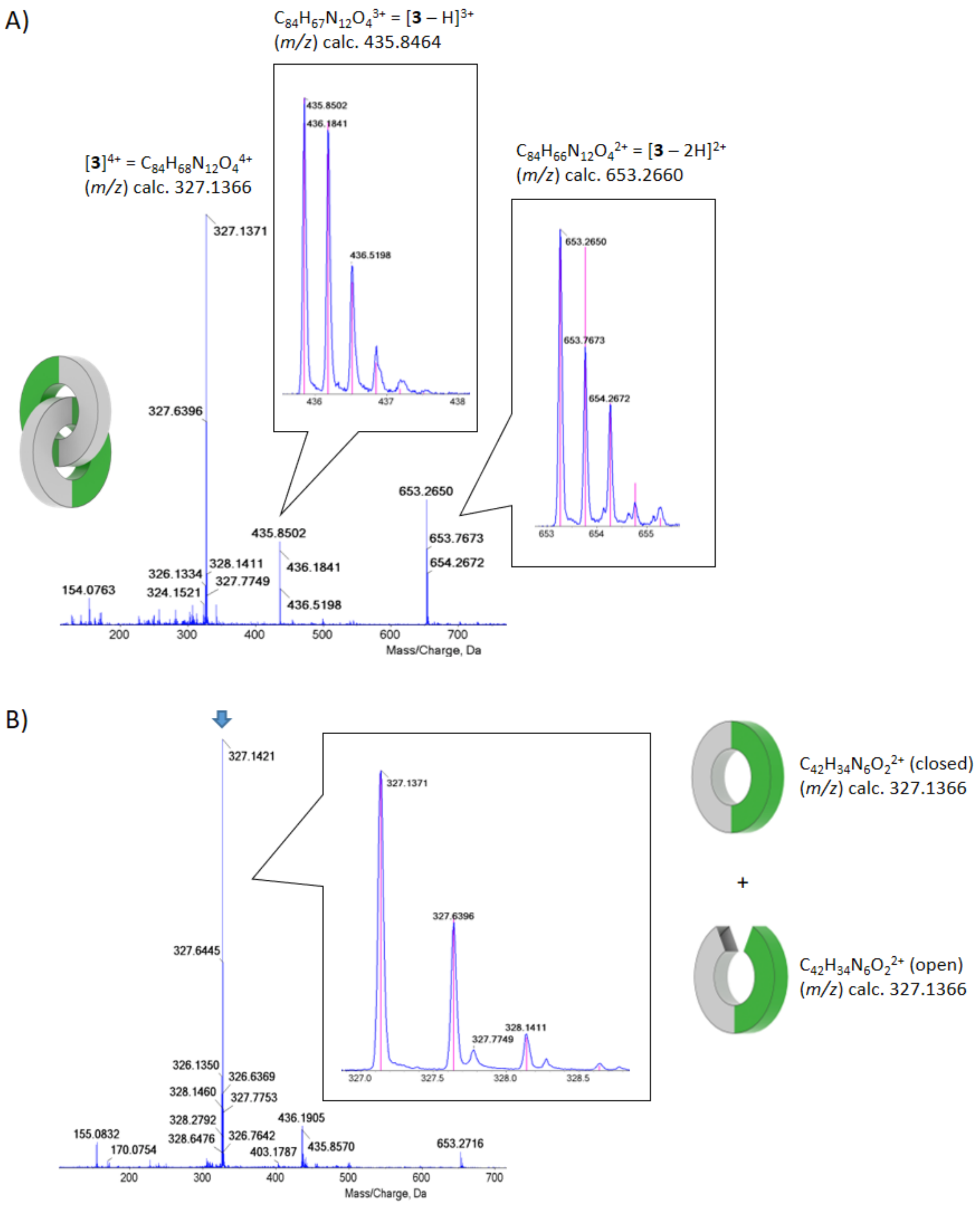

Figure S4. **A)** HR-MS and **B)** MS/MS fragmentation of [2]catenane **3** (precursor ion: *m/z* 327.1, collision energy voltages: 40 V). The fragmentation of [2]catenane **3** did not display any fragment larger than the [1+1] macrocycle. This fragmentation pattern is characteristic of a [2]catenane composed of two interlocked [1+1] macrocycles. The pink lines represent the predicted isotopic distribution.

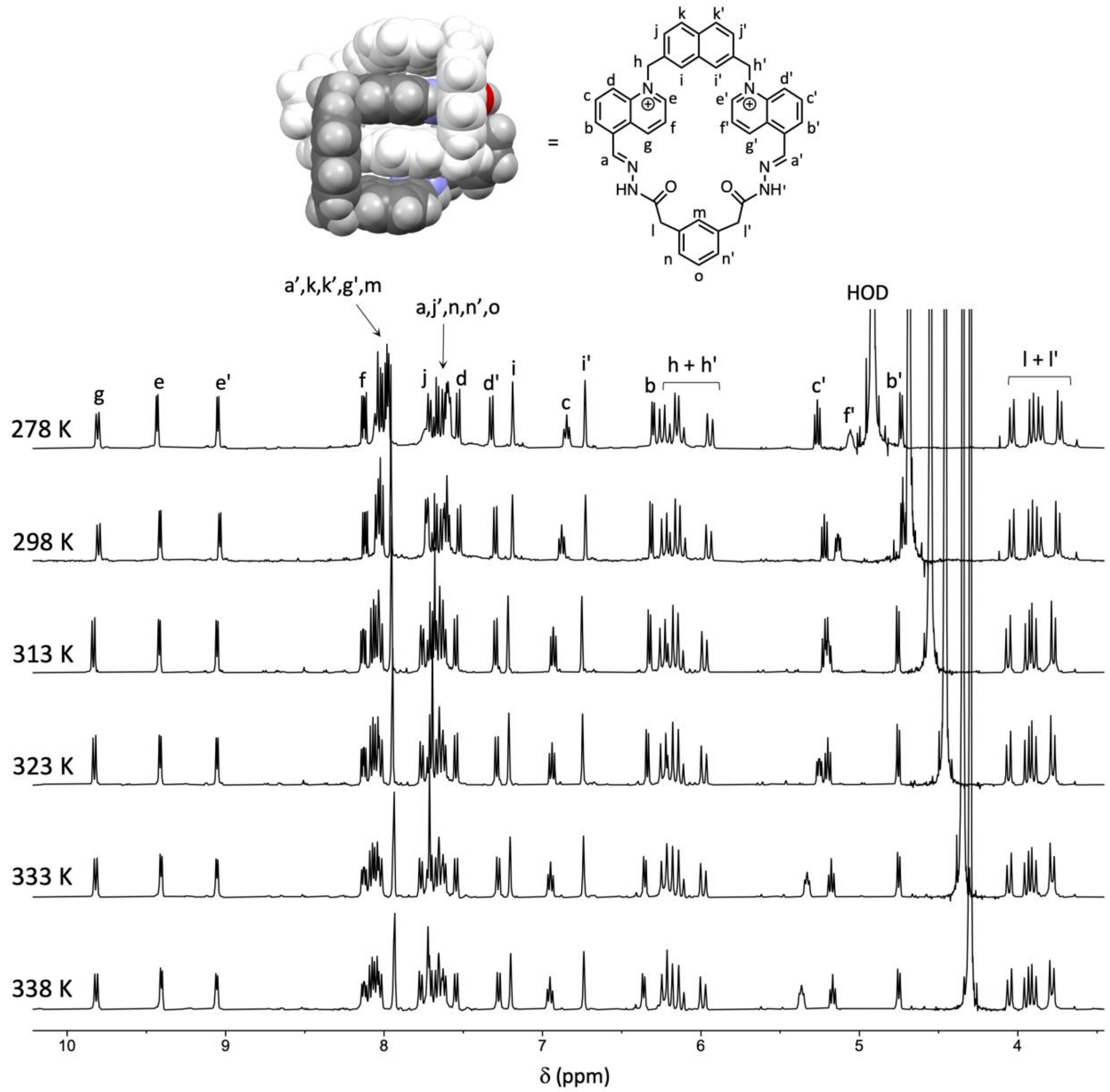

Figure S5. Variable temperature ^{1}H NMR spectra of [2]catenane **3** between 278 K and 338 K (D_2O, 500 MHz).

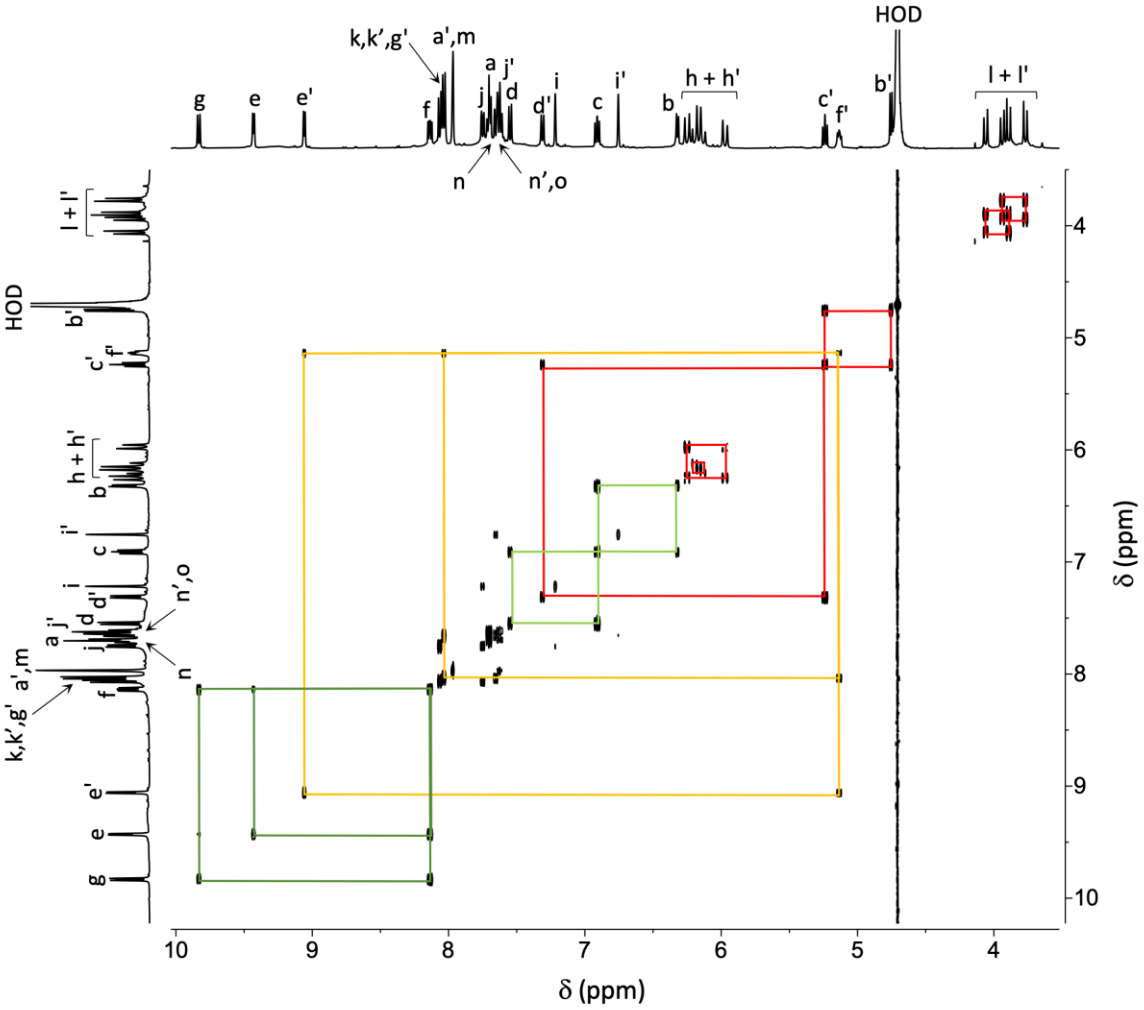

Figure S6. COSY spectrum of [2]catenane **3** (D_2O, 500 MHz, 298 K). The COSY correlations are highlighted with rectangles. A zoom on the crowded area between 6 ppm and 8 ppm is presented on the next page.

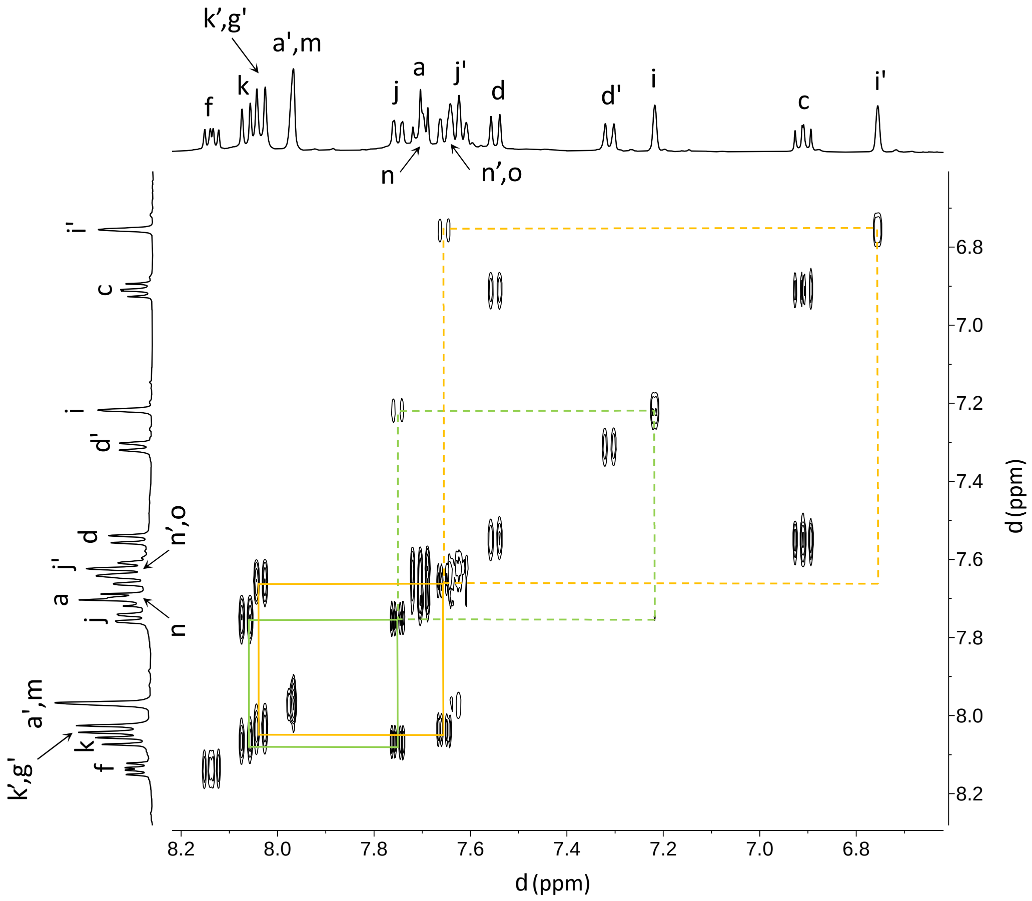

Figure S7. Partial COSY spectrum of [2]catenane **3** (D_2O, 500 MHz, 298 K). The COSY correlations are highlighted with rectangles.

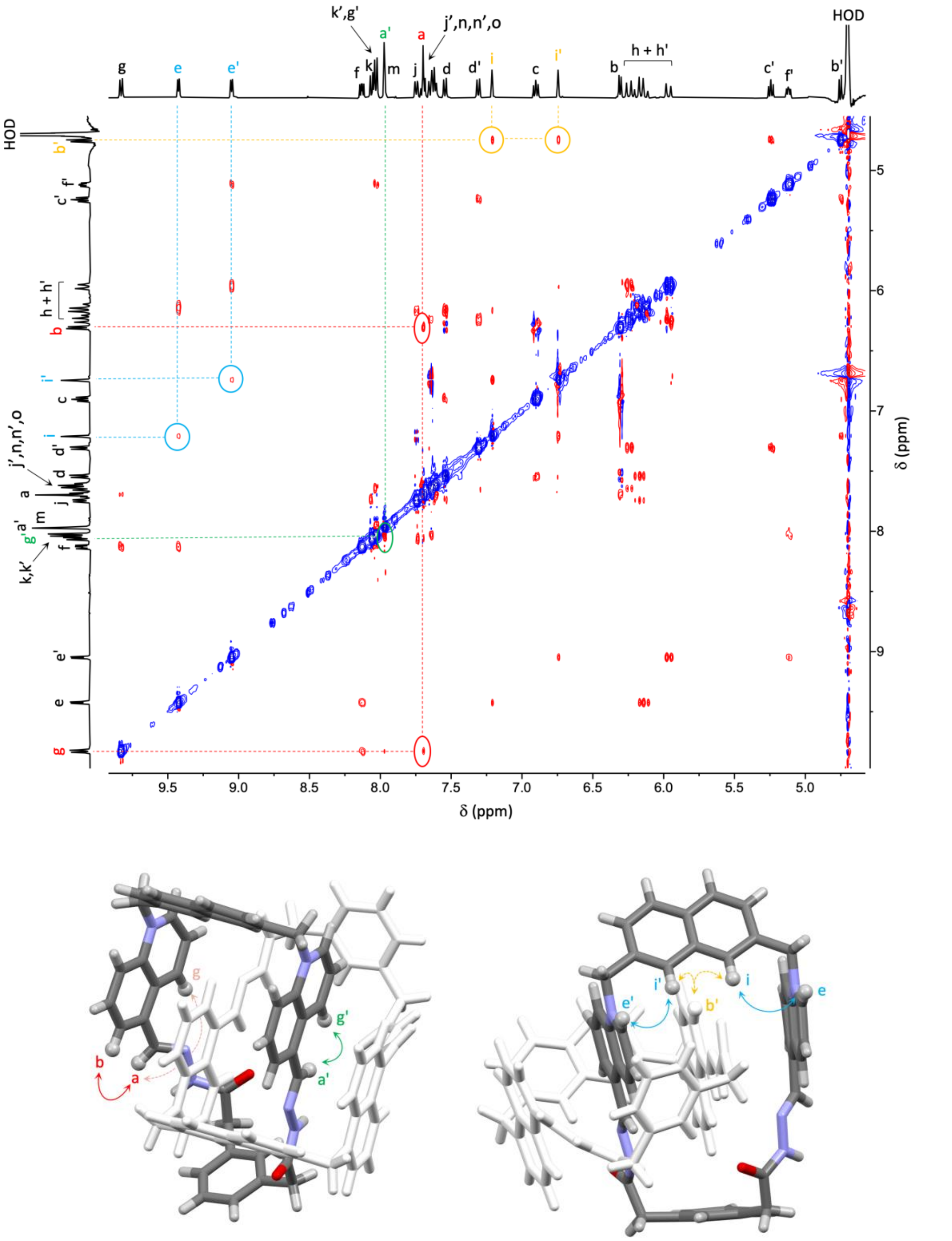

Figure S8. Partial ROESY spectrum of [2]catenane **3** (D_2O, 500 MHz, 298 K, d_8 = 400 ms). The observed correlations are consistent with the crystal structure of the [2]catenane, shown below the spectrum. The presence of the correlations **i** ↔ **b′** ↔ **i′** is particularly important since it allows for a precise determination of the relative orientation of the quinolinium units within the [2]catenane.

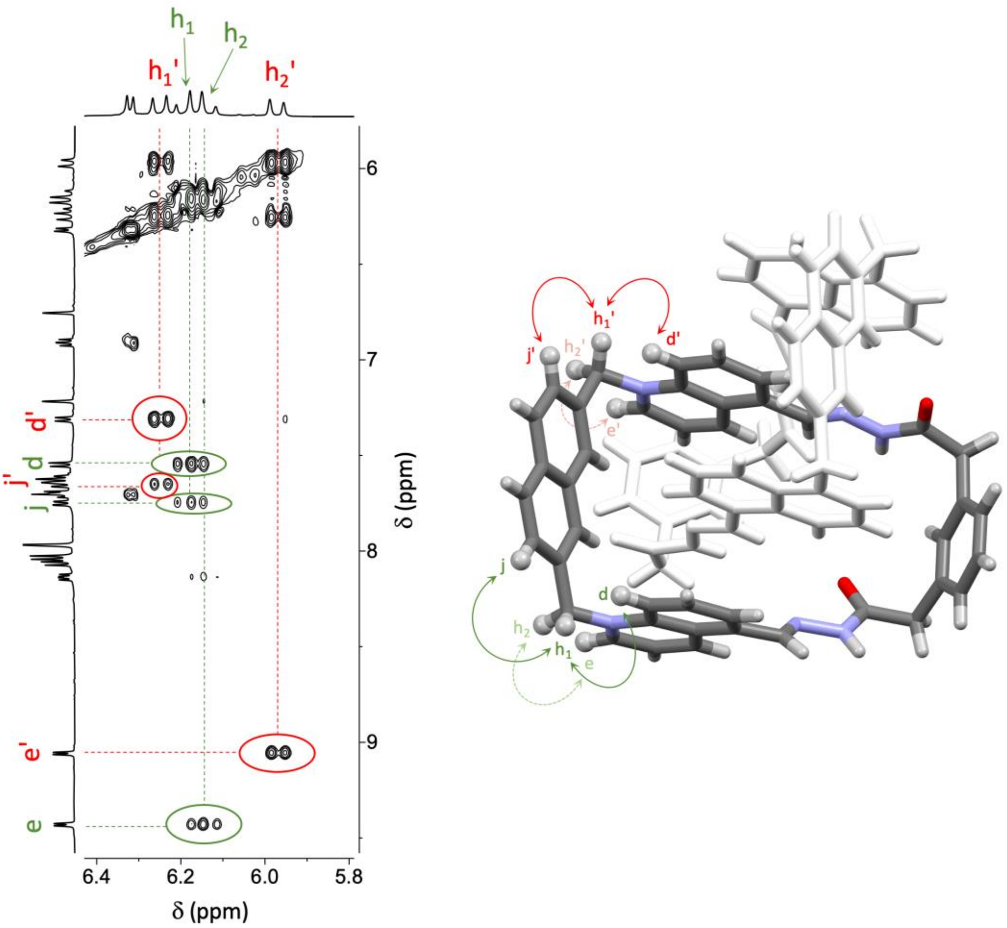

Figure S9. **A)** Partial NOESY spectrum of [2]catenane **3** (D_2O, 500 MHz, 298 K, d_8 = 300 ms). The NOE correlations highlighted with circles were used to assign the diastereotopic protons h_1, h_2, $h_1´$ and $h_2´$.

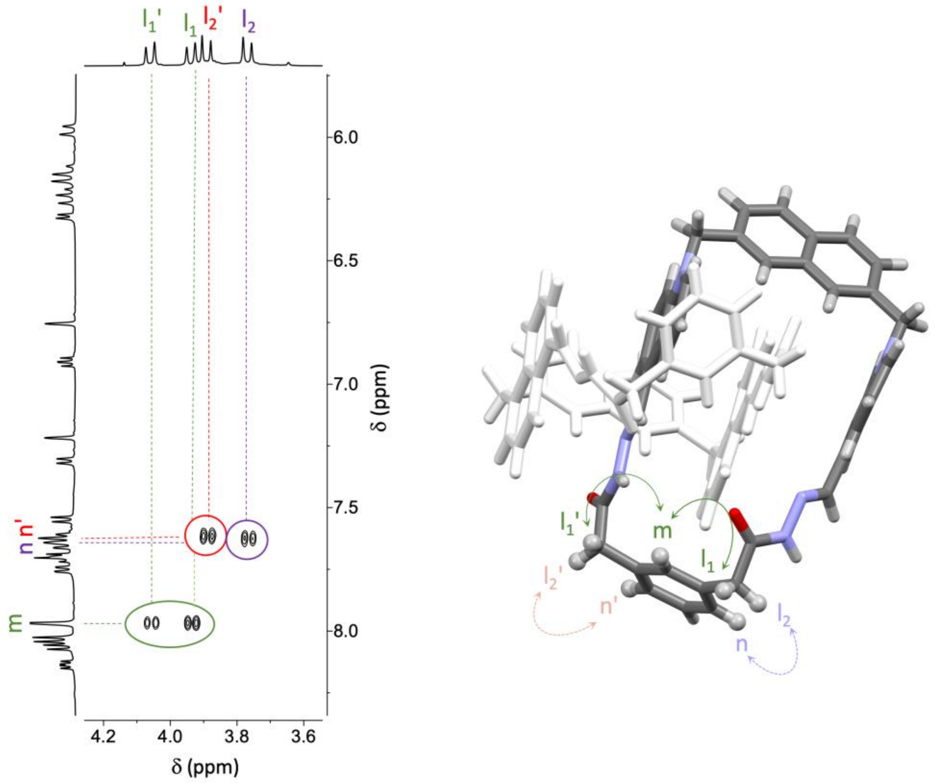

Figure S10. A) Partial NOESY spectrum of [2]catenane **3** (D_2O, 500 MHz, 298 K, d_8 = 300 ms). The NOE correlations highlighted with circles were used to assign the diastereotopic protons l_1, l_2, l_1´ and l_2´.

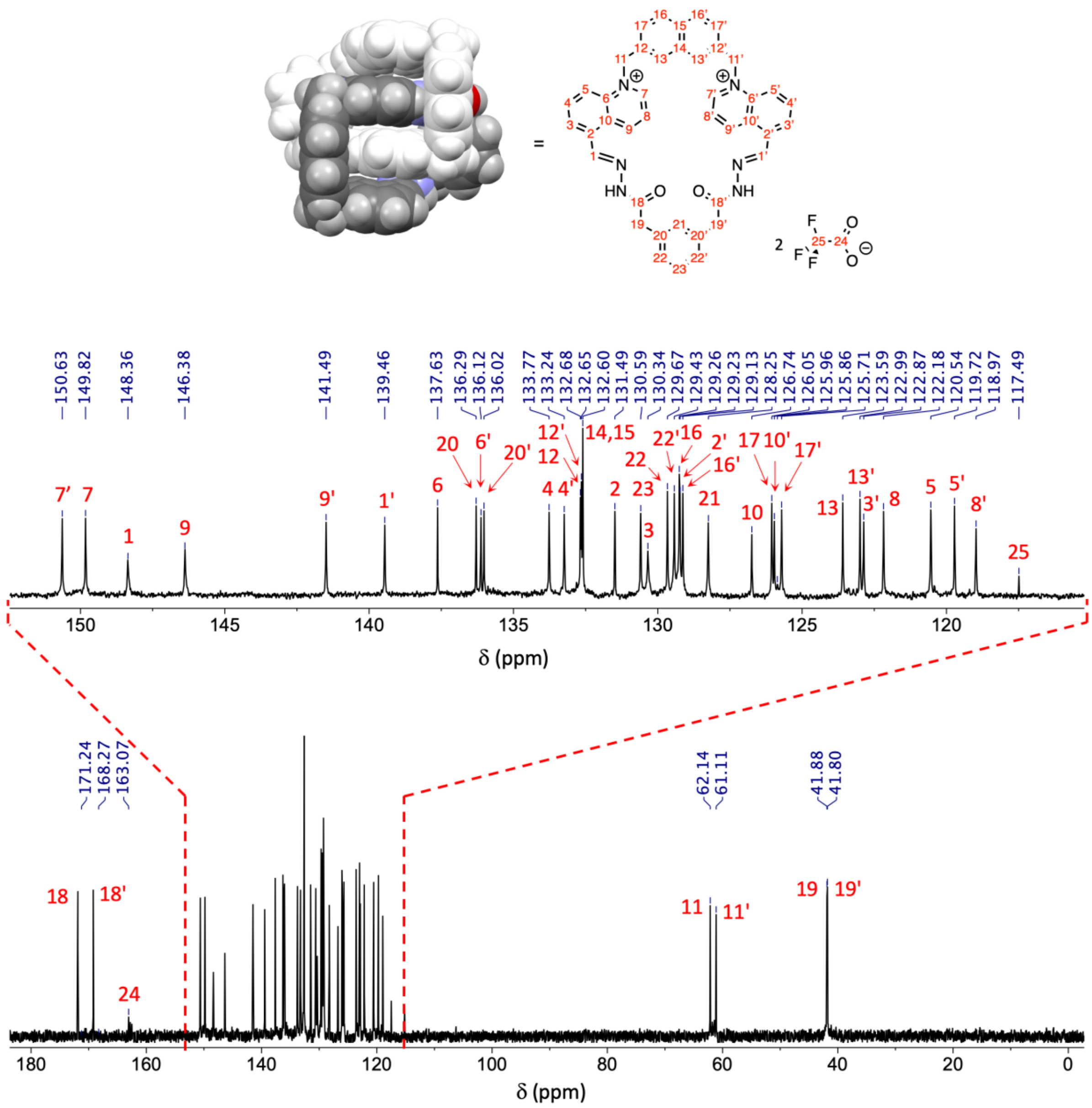

Figure S11. ^{13}C NMR spectrum of [2]catenane **3** (D_2O, 125 MHz, 298 K).

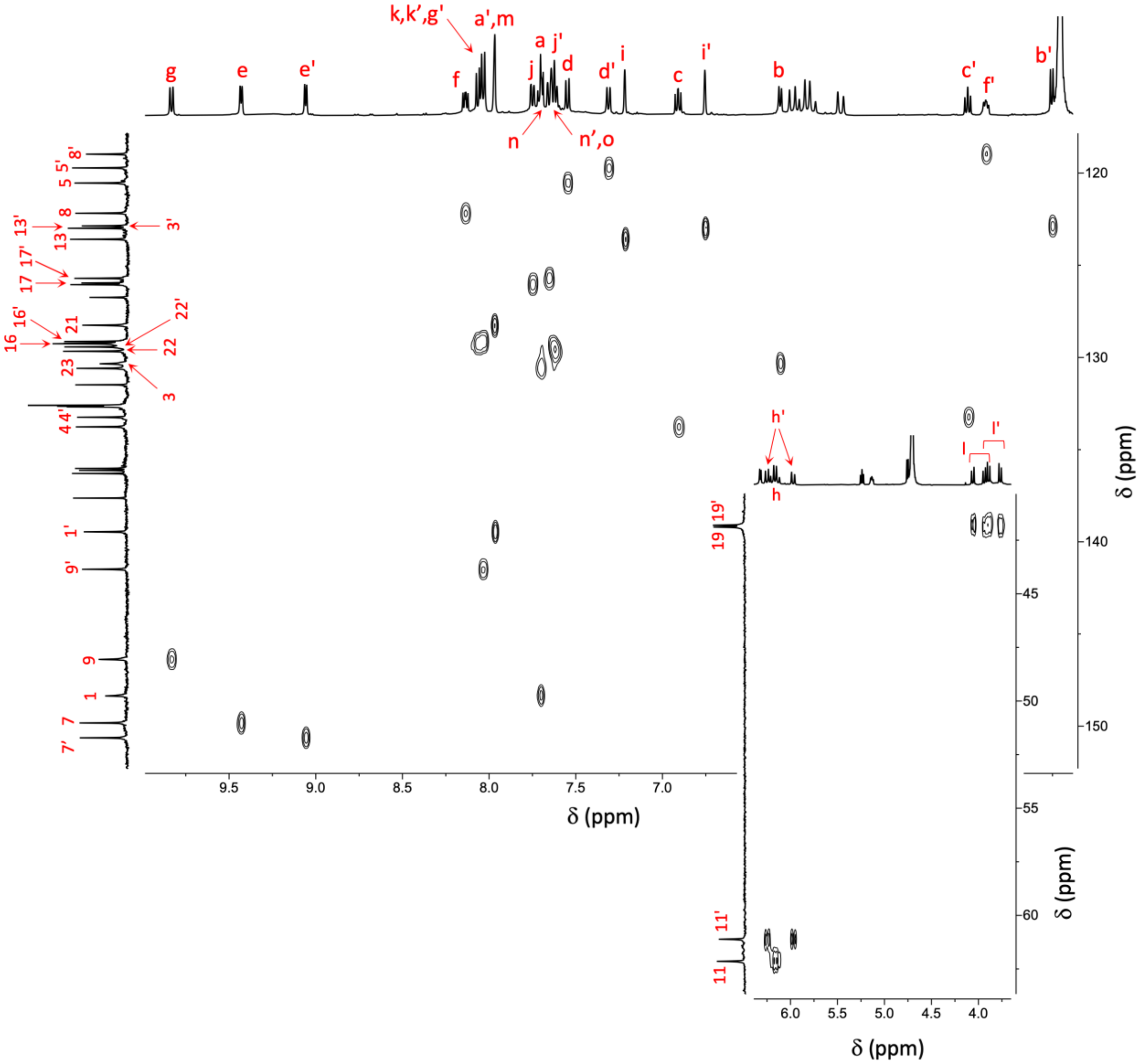

Figure S12. HSQC spectrum of [2]catenane **3** (D_2O, 298 K).

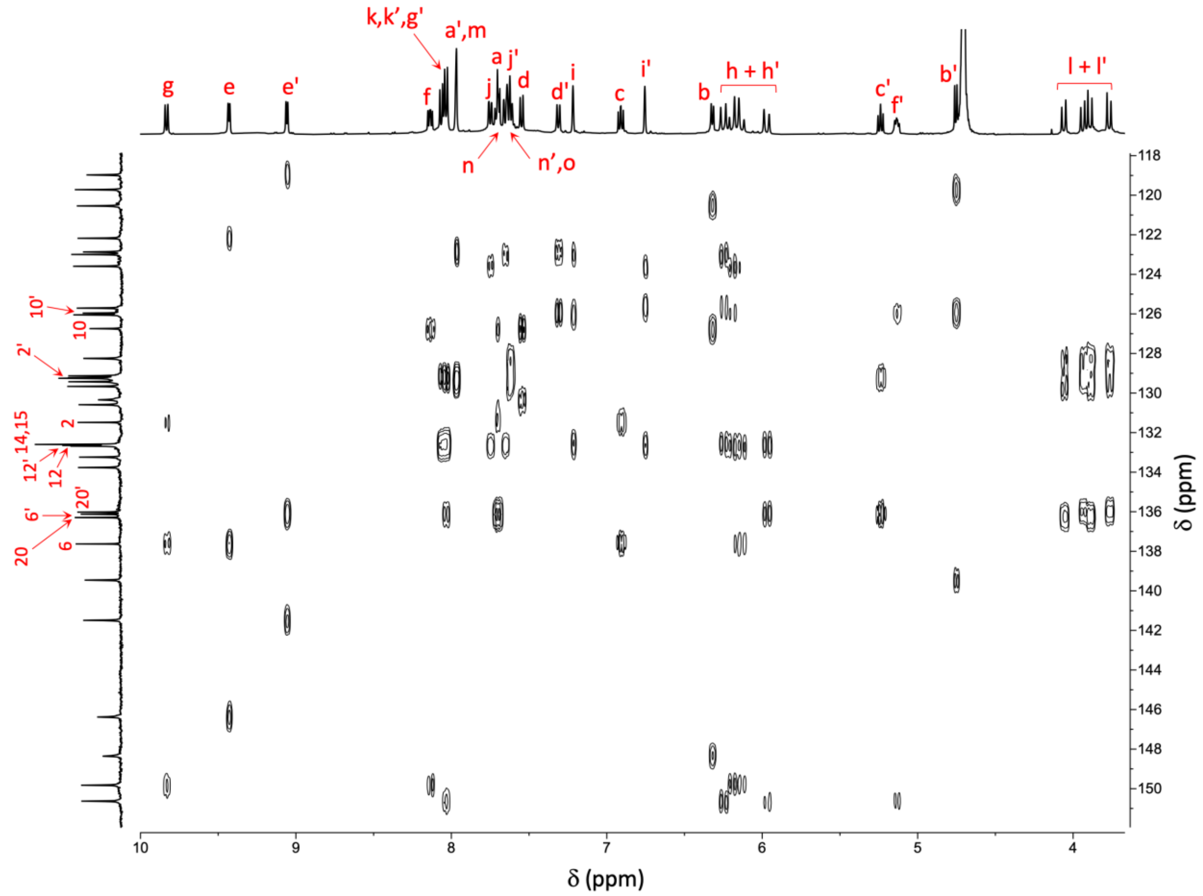

Figure S13. HMBC spectrum of [2]catenane **3** (D_2O, 298 K).

4. Determination of the thermodynamic parameters of enantiomerization of [2]catenane 3 by Exchange Spectroscopy (EXSY)

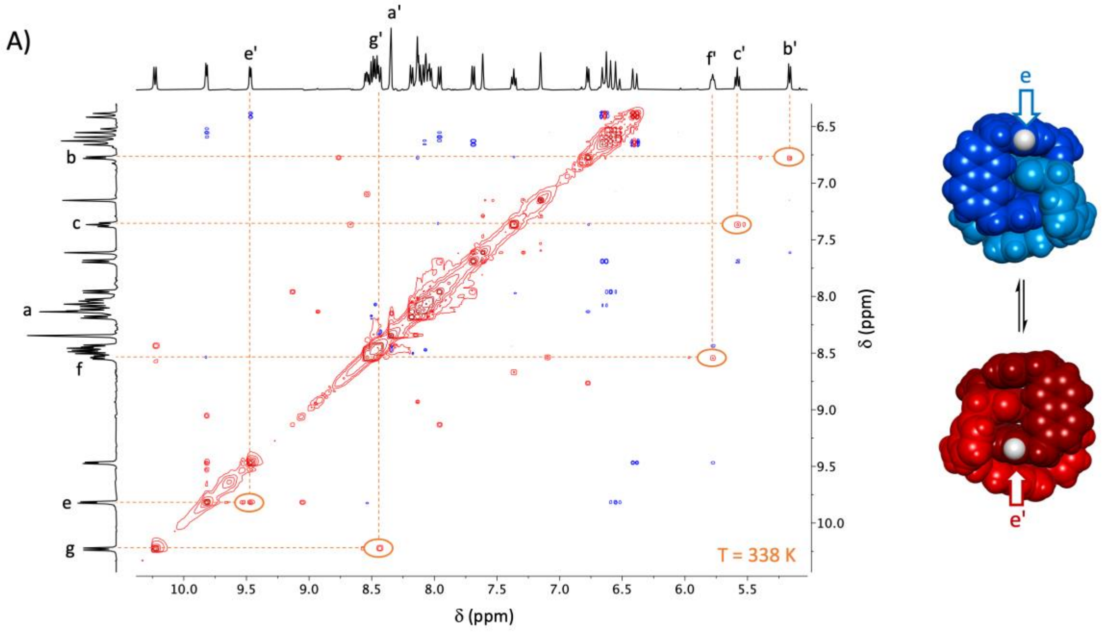

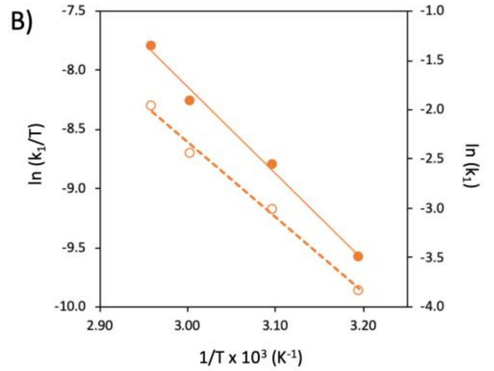

C)

		Rate constant		
T (K)	$1/T \times 10^3$ (K^{-1})	k_1 (s^{-1})	$\ln(k_1)$	$\ln(k_1/T)$
**313**	3.195	0.0216	-3.83	-9.58
**323**	3.096	0.0490	-3.02	-8.79
**333**	3.003	0.0865	-2.45	-8.26
**338**	2.959	0.1397	-1.97	-7.79
	**Kinetic parameters**	$\Delta H^{\ddagger}$ ($kJ.mol^{-1}$)	$\Delta S^{\ddagger}$ ($J.mol^{-1}.K^{-1}$)	$\Delta G^{\ddagger}$ ($kJ.mol^{-1}$)*
	(Eyring equation)	60.70	-83.17	85.48
		**Ea** ($kJ.mol^{-1}$)		** at 298 K*
	(Arrhenius equation)	63.40		

Figure S14. Determination of the thermodynamic parameters of the enantiomerization of [2]catenane **3** in pure D_2O. **A)** The partial NOESY spectrum of [2]catenane **3** (D_2O, 500 MHz, 338 K, d_8 = 300 ms) shows the presence of exchange cross-peaks between pairs of inequivalent protons (e.g. **e**↔**e′**). These signals correspond to the exchange between (*P*)-**3** and (*M*)-**3**. **B)** The thermodynamic parameters of enantiomerization were obtained by EXSY at variable temperatures (313 K - 338 K). The Eyring plot ln k_1/T against 1/T (filled circles, left axis) generated the enthalpy $\Delta H^{\ddagger}$ and the entropy $\Delta S^{\ddagger}$ of activation. The Arrhenius plot ln k_1 against 1/T (empty circles, right axis) generated the activation energy E_a. **C)** All the data is summarized in this table. The enantiomerization rates are consistent with the hypothesis that enantiomerization occurs through either mechanism of ring circumrotation or pirouetting. The enantiomerization is unlikely to occur through the opening of an acylhydrazone linkage, unthreading of the open macrocycle, re-threading and closure of the acylhydrazone linkage, since acylhydrazone bond exchange occurs at a time scale ranging from hours to days.

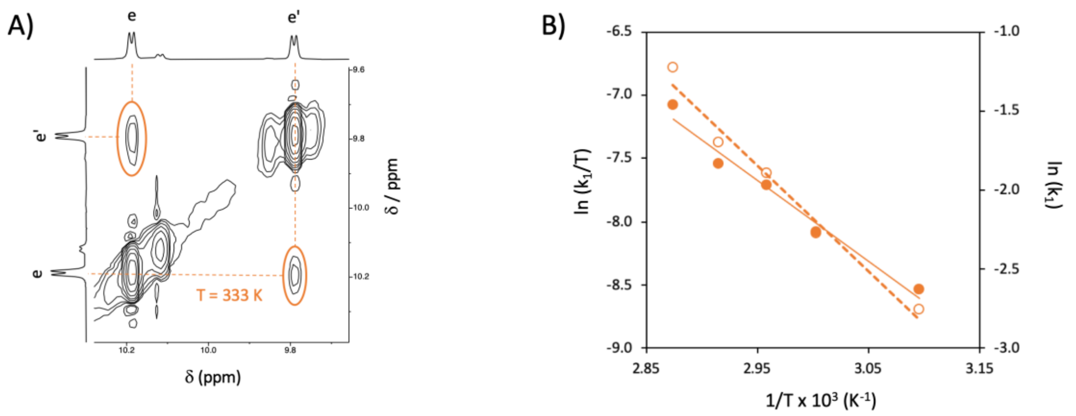

C)

T (K)	1/T x 10^3 (K^{-1})	Rate constant<br>k_1 (s^{-1})	ln(k_1)	ln(k_1/T)
**323**	3.096	0.063	-2.76	-8.54
**333**	3.003	0.102	-2.28	-8.09
**338**	2.959	0.15	-1.90	-7.72
**343**	2.915	0.181	-1.71	-7.55
**348**	2.874	0.292	-1.23	-7.08
**Kinetic parameters**		$\Delta H^‡$ (kJ.mol^{-1})	$\Delta S^‡$ (J.mol^{-1}.K^{-1})	$\Delta G^‡$ (kJ.mol^{-1})*
(Eyring equation)		52.93	-105.20	84.28
		**Ea** (kJ.mol^{-1})		** at 298 K*
(Arrhenius equation)		55.72		

Figure S15. Determination of the thermodynamic parameters of the enantiomerization of [2]catenane **3** in the solvent mixture D_2O/CD_3CN 1:1. **A)** The partial NOESY of [2]catenane **3** (D_2O/CD_3CN 1:1, 500 MHz, 333 K, d_8 = 300 ms) shows the presence of exchange cross-peaks between pairs of inequivalent protons **e**↔**e′**. **B)** The thermodynamic parameters of enantiomerization were obtained by EXSY at variable temperatures (323 K - 348 K). The Eyring plot ln k_1/T against 1/T (filled circles, left axis) generated the enthalpy $\Delta H^‡$ and the entropy $\Delta S^‡$ of activation. The Arrhenius plot ln k_1 against 1/T (empty circles, right axis) generated the activation energy E_a. **C)** All the data is summarized in this table.

5. Diastereoselective amplification and characterization of the diastereomeric complex (*P*)-3·(*R*)-4

The equilibria involved in the amplification of (*P*)-**3**·(*R*)-**4** from the racemic mixture of the two enantiomeric co-conformers are:

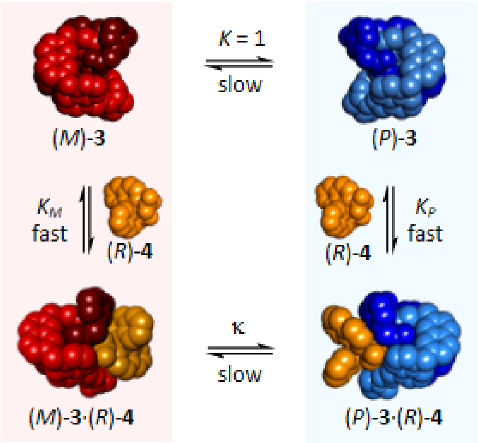

[P] is the concentration of free (*P*)-**3**
[M] is the concentration of free (*M*)-**3**
[**4**] is the concentration of free (*R*)-**4**
[P·**4**] is the concentration of the complex (*P*)-**3**·(*R*)-**4**
[M·**4**] is the concentration of the complex (*M*)-**3**·(*R*)-**4**
$[C]_t$ is the total concentration of [2]catenane
$[\mathbf{4}]_t$ is the total concentration of (*R*)-**4**

$$[M] = [P]$$
$$[M{\cdot}\mathbf{4}] = K_M\,[M]\,[\mathbf{4}]$$
$$[P{\cdot}\mathbf{4}] = K_P\,[P]\,[\mathbf{4}]$$
$$1/\kappa = \frac{[M{\cdot}\mathbf{4}]}{[P{\cdot}\mathbf{4}]}$$

The parameter measured during the titration is the diastereomeric excess

$$\text{d.e.} = \frac{[P] + [P{\cdot}\mathbf{4}] - [M] - [M{\cdot}\mathbf{4}]}{[C]_t} = \frac{[P{\cdot}\mathbf{4}] - [M{\cdot}\mathbf{4}]}{[C]_t} = \frac{[P{\cdot}\mathbf{4}]}{[C]_t}\,(1 - 1/\kappa) \qquad (1)$$

The mass balance of [2]catenane is:

$$[C]_t = [P] + [M] + [P{\cdot}\mathbf{4}] + [M{\cdot}\mathbf{4}]$$
$$[C]_t = 2\,[P] + (1 + 1/\kappa)\,[P{\cdot}\mathbf{4}]$$
$$[C]_t = \frac{2\,[P{\cdot}\mathbf{4}]}{K_P\,[R]} + (1 + 1/\kappa)\,[P{\cdot}\mathbf{4}]$$
$$[C]_t = [P{\cdot}\mathbf{4}]\left(\frac{2}{K_P\,[R]} + (1 + 1/\kappa)\right) \qquad (2)$$

The mass balance of chiral anion (*R*)-**4** is:

$$[\mathbf{4}]_t = [\mathbf{4}] + [P{\cdot}\mathbf{4}] + [M{\cdot}\mathbf{4}]$$
$$[\mathbf{4}]_t = [\mathbf{4}] + (1 + 1/\kappa)\,[P{\cdot}\mathbf{4}]$$
$$[\mathbf{4}] = [\mathbf{4}]_t - (1 + 1/\kappa)\,[P{\cdot}\mathbf{4}] \qquad (3)$$

Combining equations *(2)* and *(3)* yields the following equation:

$$[C]_t = [P{\cdot}\mathbf{4}]\left(\frac{2}{K_P\,[\mathbf{4}]_t - K_P\,(1 + 1/\kappa)\,[P{\cdot}\mathbf{4}]} + (1 + 1/\kappa)\right)$$

$$\frac{[C]_t}{[P{\cdot}\mathbf{4}]} = \frac{2}{K_P\,[\mathbf{4}]_t - K_P\,(1 + 1/\kappa)\,[P{\cdot}\mathbf{4}]} + (1 + 1/\kappa)$$

$$\frac{[C]_t}{[P{\cdot}\mathbf{4}]} - (1 + 1/\kappa) = \frac{2}{K_P\,[\mathbf{4}]_t - K_P\,(1 + 1/\kappa)\,[P{\cdot}\mathbf{4}]}$$

$$\frac{[C]_t - (1 + 1/\kappa)\,[P{\cdot}\mathbf{4}]}{[P{\cdot}\mathbf{4}]} = \frac{2}{K_P\,[\mathbf{4}]_t - K_P\,(1 + 1/\kappa)\,[P{\cdot}\mathbf{4}]}$$

$$\left(K_P\,[\mathbf{4}]_t - K_P\,(1 + 1/\kappa)\,[P{\cdot}\mathbf{4}]\right)\left([C]_t - (1 + 1/\kappa)\,[P{\cdot}\mathbf{4}]\right) = 2\,[P{\cdot}\mathbf{4}]$$

$$K_P\,[\mathbf{4}]_t\,[C]_t - K_P\,[\mathbf{4}]_t\,(1 + 1/\kappa)\,[P{\cdot}\mathbf{4}] - [C]_t\,K_P\,(1 + 1/\kappa)\,[P{\cdot}\mathbf{4}] + K_P\,(1 + 1/\kappa)^2\,[P{\cdot}\mathbf{4}]^2 = 2\,[P{\cdot}\mathbf{4}]$$

$$K_P\,(1 + 1/\kappa)^2\,[P{\cdot}\mathbf{4}]^2 - \left(2 + K_P\,[\mathbf{4}]_t\,(1 + 1/\kappa) + K_P\,[C]_t\,(1 + 1/\kappa)\right)[P{\cdot}\mathbf{4}] + K_P\,[\mathbf{4}]_t\,[C]_t = 0$$

$$K_P\,(1 + 1/\kappa)^2\,[P{\cdot}\mathbf{4}]^2 - \left(2 + K_P\,(1 + 1/\kappa)\,([\mathbf{4}]_t + [C]_t)\right)[P{\cdot}\mathbf{4}] + K_P\,[\mathbf{4}]_t\,[C]_t = 0$$

$$(1 + 1/\kappa)^2\,[P{\cdot}\mathbf{4}]^2 - \left(2/K_P + (1 + 1/\kappa)([\mathbf{4}]_t + [C]_t)\right)[P{\cdot}\mathbf{4}] + [\mathbf{4}]_t\,[C]_t = 0$$

$$[P{\cdot}\mathbf{4}] = \frac{2/K_P + (1 + 1/\kappa)\,([\mathbf{4}]_t + [C]_t) - \sqrt{\left(2/K_P + (1 + 1/\kappa)\,([\mathbf{4}]_t + [C]_t)\right)^2 - 4\,[\mathbf{4}]_t\,[C]_t\,(1 + 1/\kappa)^2}}{2\,(1 + 1/\kappa)^2}$$

Given that $\text{d.e.} = \dfrac{[P{\cdot}\mathbf{4}]}{[C]_t}\,(1 - 1/\kappa)$

$$\text{d.e.} = \frac{2/K_P + (1 + 1/\kappa)\,([\mathbf{4}]_t + [C]_t) - \sqrt{\left(2/K_P + (1 + 1/\kappa)\,([\mathbf{4}]_t + [C]_t)\right)^2 - 4\,[\mathbf{4}]_t\,[C]_t\,(1 + 1/\kappa)^2}}{2\,[C]_t\,(1 + 1/\kappa)^2}\,(1 - 1/\kappa)$$

This equation was used to fit the data generated by the CD titration and to obtain the values of K_P.

The value of κ was calculated from the value d.e._{max} measured from the NMR titration. The combination of equations *(1)* and *(2)* yields the following equation:

$$\text{d.e.} = \frac{(1 - 1/\kappa)}{\left(\dfrac{2}{K_P\,[\mathbf{4}]} + (1 + 1/\kappa)\right)}$$

Given that $K_P = \dfrac{[P{\cdot}\mathbf{4}]}{[P]\,[\mathbf{4}]}$, the term $\dfrac{2}{K_P\,[\mathbf{4}]}$ can be replaced by $\dfrac{2}{K_P\,[\mathbf{4}]} = \dfrac{2\,[P]}{[P{\cdot}\mathbf{4}]}$

At saturation $\frac{2\,[P]}{[P\cdot\mathbf{4}]} \rightarrow 0$ and $d.e. \rightarrow d.e._{max} = \frac{(1-1/\kappa)}{(1+1/\kappa)}$

We measured that $1/\kappa = \frac{(1-d.e._{max})}{(1+d.e._{max})} = 0.081$ or $\kappa = 12.34$

The difference of Gibbs free energy of the two diastereomeric complexes at 298 K is

$\Delta G° = -R \cdot T \cdot \ln\kappa = 6.2\ kJ \cdot mol^{-1}$

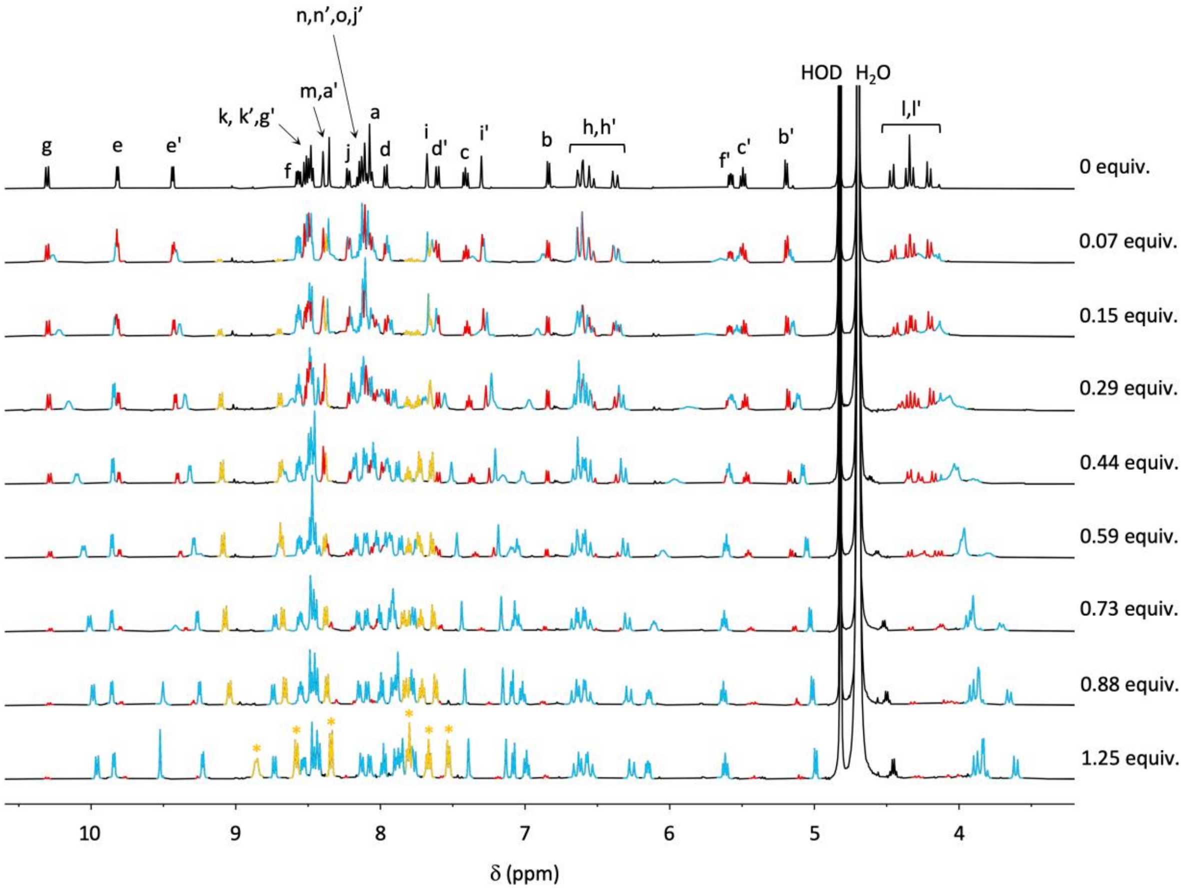

Figure S16. ^{1}H NMR spectra of [2]catenane **3** (1.13 mM, D_2O/CD_3CN 1:1, 500 MHz, 298 K) in the presence of 0–1.25 equiv. of disulfonate (*R*)-**4**. The signals colored in blue correspond to the (*P*)-catenane. More precisely, the blue signals correspond to the average signals for the unbound and the bound forms of the (*P*)-catenane, (*P*)-**3** and (*P*)-**3**·(*R*)-**4**, which are exchanging rapidly on the NMR timescale. The signals colored in red correspond to the (*M*)-catenane. More precisely, the red signals correspond to the average signals for the unbound and the bound forms of the (*M*)-catenane, (*M*)-**3** and (*M*)-**3**·(*R*)-**4**, which are exchanging rapidly on the NMR timescale. The signals colored in yellow and marked with a star correspond to disulfonate (*R*)-**4**.

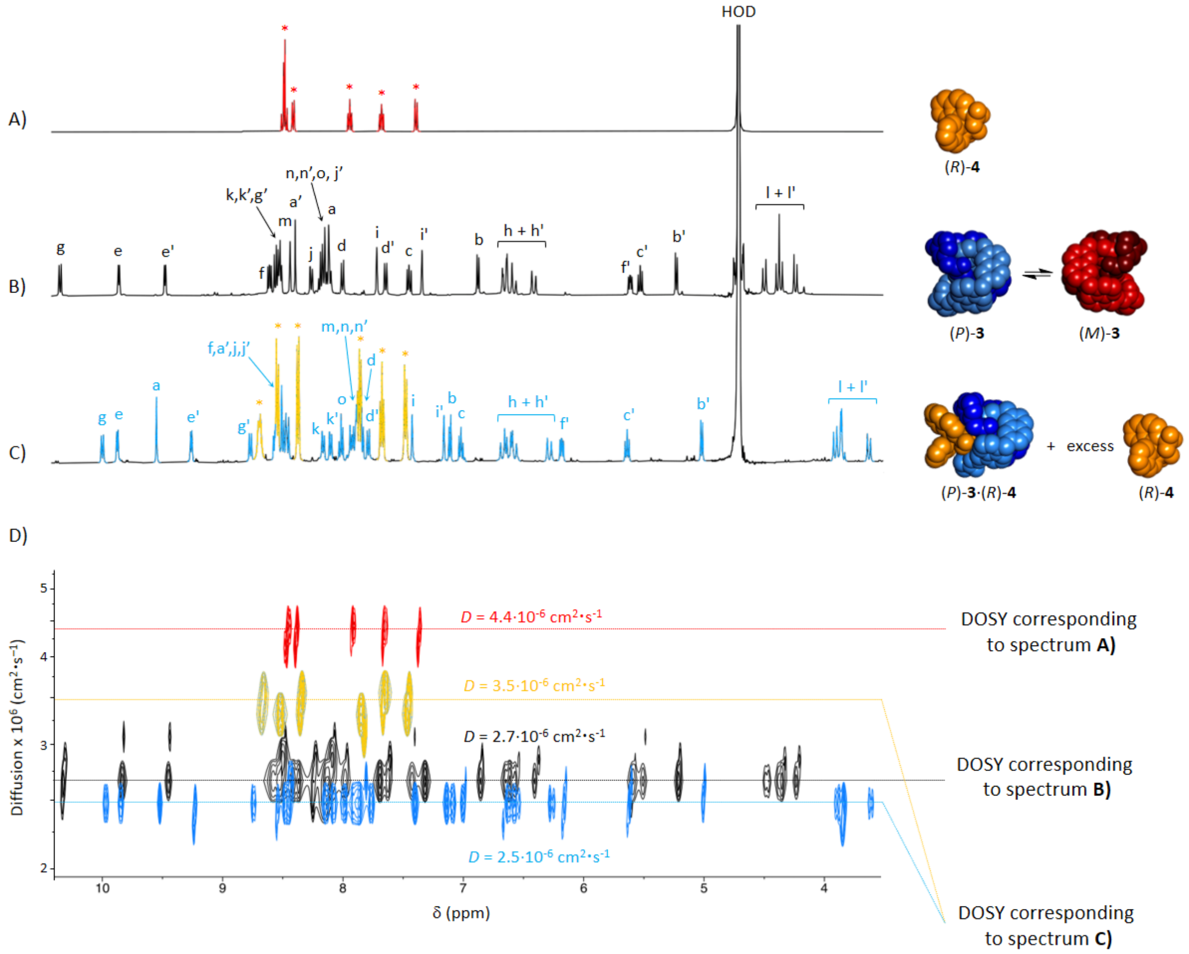

Figure S17. Comparison between the ^{1}H NMR spectra of **A)** disulfonate (*R*)-**4** alone (2.26 mM); **B)** the racemic mixture of [2]catenane **3** alone (1.13 mM) and ; **C)** the diastereomeric complex (*P*)-**3**·(*R*)-**4** (1.13 mM, colored in blue) obtained after addition of 2 equiv. of (*R*)-**4** (2.26 mM, colored in yellow and marked with a star). Panel **D)** shows the overlap of the corresponding diffusion ordered spectra. All these spectra were recorded in the same conditions (D_2O/CD_3CN 1:1, 500 MHz, 298 K).

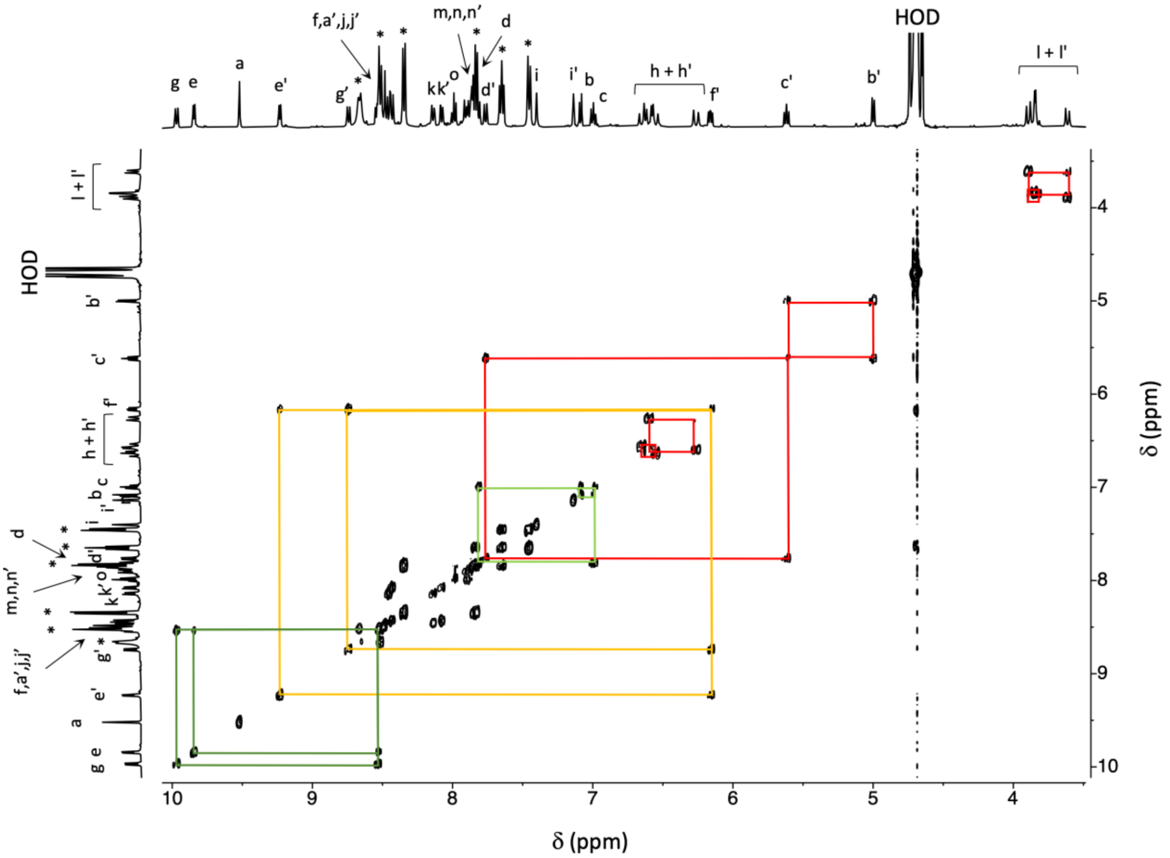

Figure S18. COSY spectrum (D_2O/CD_3CN 1:1, 500 MHz, 298 K) of the diastereomeric complex (*P*)-**3**·(*R*)-**4** obtained after addition of 2 equiv. of (*R*)-**4**. The COSY correlations are highlighted with rectangles. The signals marked with a star belong to (*R*)-**4**. Unfortunately, the NOESY spectrum of the diastereomeric complex (*P*)-**3**·(*R*)-**4** (data not shown) did not show any noe correlation between the protons of the [2]catenane and those of the disulfonate.

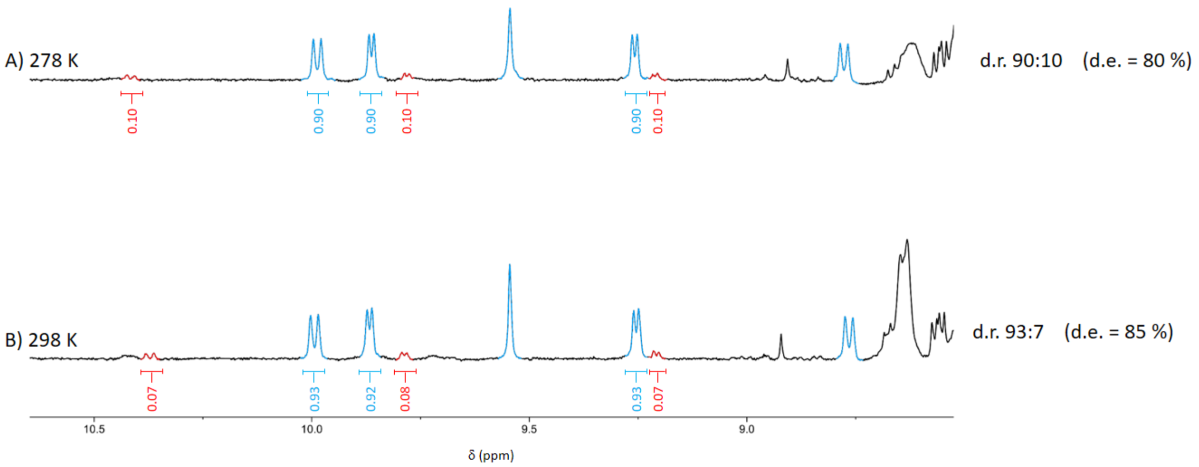

Figure S19. Partial ^{1}H NMR spectra of the [2]catenane **3** (1.13 mM, D_2O/CD_3CN 1:1, 500 MHz) after addition of 2 equiv. of (*R*)-**4** (2.26 mM) at **A)** 278 K and **B)** 298 K. The diastereomeric excess d.e.$_{max}$ is not improved at lower temperature.

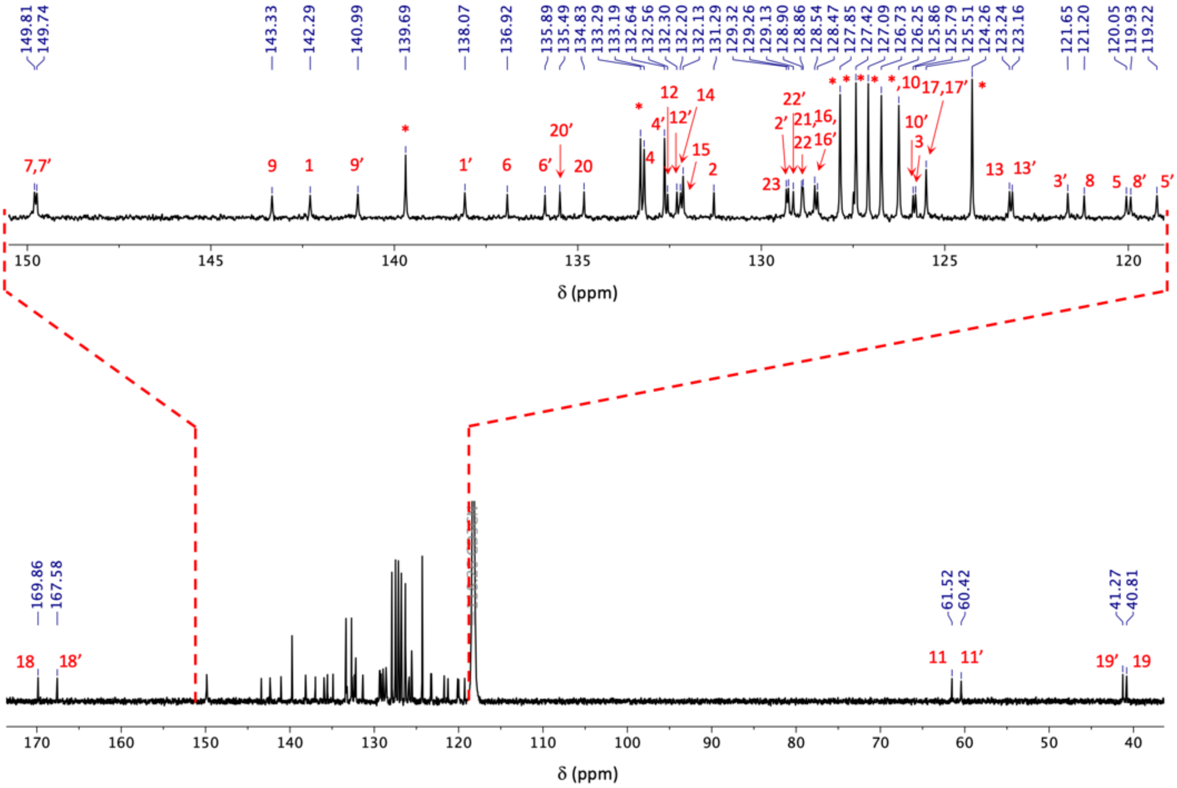

Figure S20. ^{13}C NMR spectrum (D_2O/CD_3CN 1:1, 500 MHz, 298 K) of the diastereomeric complex (*P*)-**3**·(*R*)-**4** obtained after addition of 2 equiv. of (*R*)-**4**. The signals marked with a star belong to (*R*)-**4**.

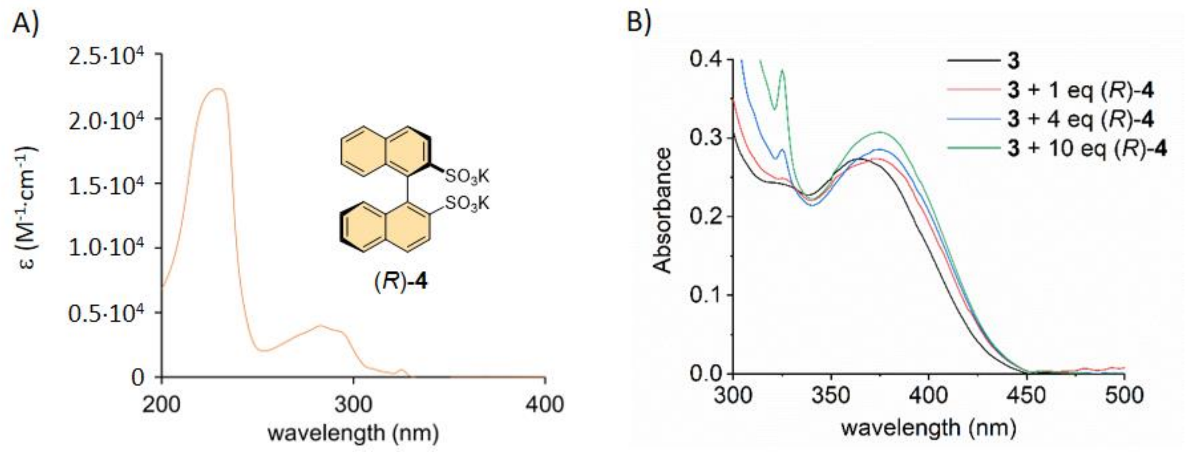

Figure S21. **A)** UV-visible spectrum of (*R*)-**4** (H_2O/CH_3CN 1:1). **B)** UV-visible spectra of [2]catenane **3** in the presence of 0–10 equiv. of (*R*)-**4** (H_2O/CH_3CN 1:1).

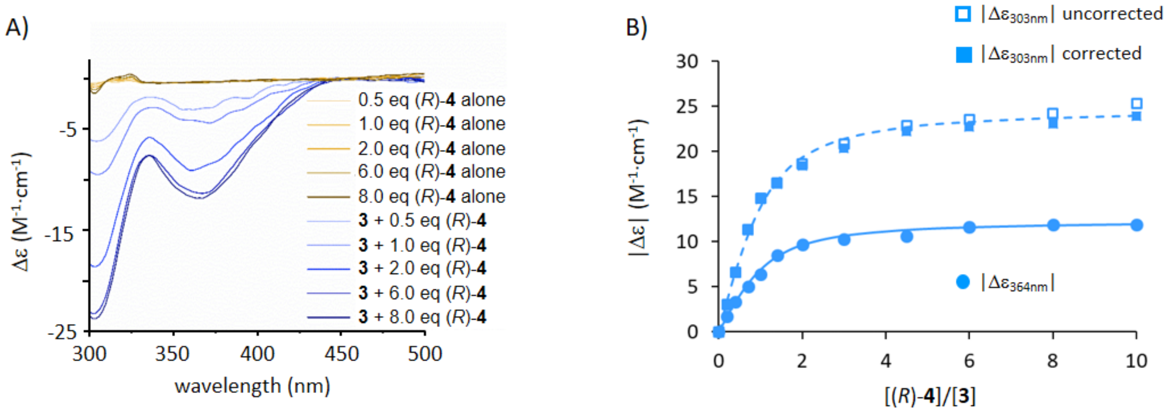

Figure S22. **A)** CD spectra of [2]catenane **3** (38 µM, H_2O/CH_3CN 1:1) in the presence of 0–8 equiv. of (*R*)-**4**. The orange traces show the control spectra recorded with (*R*)-**4** alone. Overall, the CD contribution of (*R*)-**4** is negligible at 364 nm and relatively weak at 303 nm. **B)** Graph showing the evolution of Δε in the course of the titration. Here we are comparing the values of $\Delta\varepsilon_{303nm}$ directly measured (empty squares), $\Delta\varepsilon_{303nm}$ corrected (after the weak CD contribution from (*R*)-**4** was subtracted, full squares) and $\Delta\varepsilon_{364nm}$ (full circles). Fitting $\Delta\varepsilon_{303nm}$ corrected and $\Delta\varepsilon_{364nm}$ as a function of the number of equivalents of (*R*)-**4** gave consistent results. Only the data $\Delta\varepsilon_{364nm}$ = f ([(*R*)-**4**]/[**3**]) is showed in the manuscript (Figure 7, inset).

6. Crystallography

A crystal was selected and mounted in a cryoloop (Hampton Research) using fomblin oil. Manipulation of the crystal was done at the top of a half-filled nitrogen vessel to protect the crystals from degradation.

Data were collected on an Agilent supernova dual source diffractometer equipped with an Atlas detector, using Cu Kα radiation. Data reduction was carried out in the crysalis Pro Software.

Structure solution was made using dual space methods in the SHELXT [S2] software. Further refinements of the model were carried out using full matrix least-square on F2 in SHELXL [S2] within the Olex2 [S3] software. High angle data were weak so that the data were cut at a resolution of 0.9 Å.

A representation of the asymmetric units is depicted in Figure 23. Apart from the [2]catenane, four disordered hexafluorophosphate anion are present. They were refined using two or three rigid bodies each. Restraints and constraints were applied on anisotropic displacement parameters.

Two water molecules, each disordered over two positions hydrogen bond to the [2]catenane. The position of the hydrogen atoms of these water molecules were assigned considering remaining density in the residual Fourier map and potential hydrogen bonds. Data are not good enough to unambiguously confirm the position of theses hydrogen atoms. Restraints were applied on anisotropic displacement parameters. Finally, an attempt was made to model the solvent. Partially occupied disordered acetonitrile molecules and water molecules were added to the model. Hydrogen atoms were not added to the very disordered partially occupied water molecules. Constraints/restraints were used on bond lengths and displacement parameters. This solvent model is highly approximate.

Details on the refinement can be found on the next page.

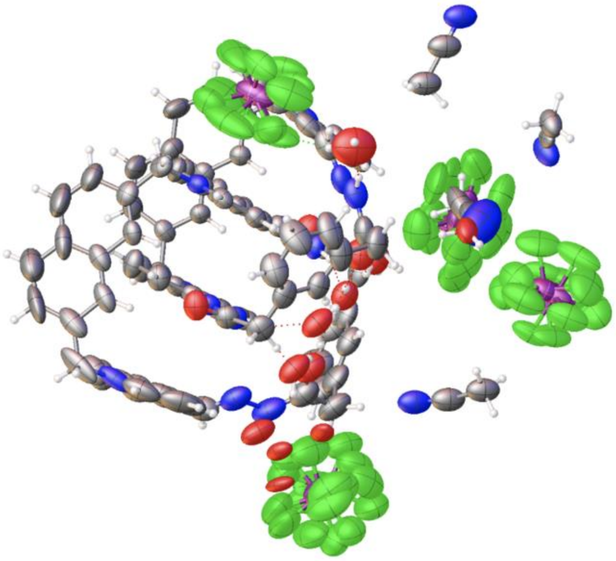

Figure S23. View of the asymmetric unit. Displacement ellipsoids are depicted at 50 % probability level.

Crystal data and structure refinement

Identification code	kenji_last
Empirical formula	C86.97 H79.32 F23.95 N13.49 O8.43 P3.99
Formula weight	2027.02
Temperature	180.00(10) K
Wavelength	1.54184 Å
Crystal system	Orthorhombic
Space group	$Pna2_1$
Unit cell dimensions	a = 25.6044(5) Å a = 90°
	b = 14.24157(15) Å b = 90°
	c = 26.3363(4) Å g = 90°
Volume	9603.5(2) $Å^3$
Z	4
Density (calculated)	1.402 Mg/m^3
Absorption coefficient	1.674 mm^{-1}
F(000)	4154
Crystal size	0.345 x 0.163 x 0.094 mm^3
Theta range for data collection	3.356 to 58.929°
Index ranges	-24<=h<=28, -15<=k<=6, -29<=l<=29
Reflections collected	32267
Independent reflections	13188 [R(int) = 0.0220]
Completeness to theta = 58.929°	100.0 %
Absorption correction	Analytical
Max. and min. transmission	0.879 and 0.636
Refinement method	Full-matrix least-squares on F2
Data / restraints / parameters	13188 / 842 / 1510
Goodness-of-fit on F2	1.036
Final R indices [I>2sigma(I)]	R1 = 0.0831, wR2 = 0.2207
R indices (all data)	R1 = 0.0928, wR2 = 0.2360
Absolute structure parameter	0.56(5)
Extinction coefficient	n/a
Largest diff. peak and hole	0.627 and -0.327 e. $Å^{-3}$

7. DFT calculations

The geometries included in this study have been fully optimized at the BP86-D3/def2-TZVP level of theory using the program TURBOMOLE version 7.0. [S4] For the calculations we have used the BP86 functional with the latest available correction for dispersion (D3). [S5] In order to reproduce solvent effects, we have used the conductor-like screening model COSMO, [S6] which is a variant of the dielectric continuum solvation models. [S7] We have used water or methanol as solvent. [S8]

The optimized complexes of the diastereomeric complexes (*P*)-**3**·(*R*)-**4** and (*M*)-**3**·(*R*)-**4** are shown in Figure S24. It can be observed that complex (*P*)-**3**·(*R*)-**4** exhibit four directional H-bonds that are significantly shorter than those observed in (*M*)-**3**·(*R*)-**4**. Moreover, the calculations show that (*P*)-**3**·(*R*)-**4** is 6.7 kcal/mol more stable thermodynamically than (*M*)-**3**·(*R*)-**4**. This value is higher than the experimental value, presumably because the solvent conditions used in the calculations does not exactly mimic the experimental solvent conditions (H_2O/CH_3CN 1:1).

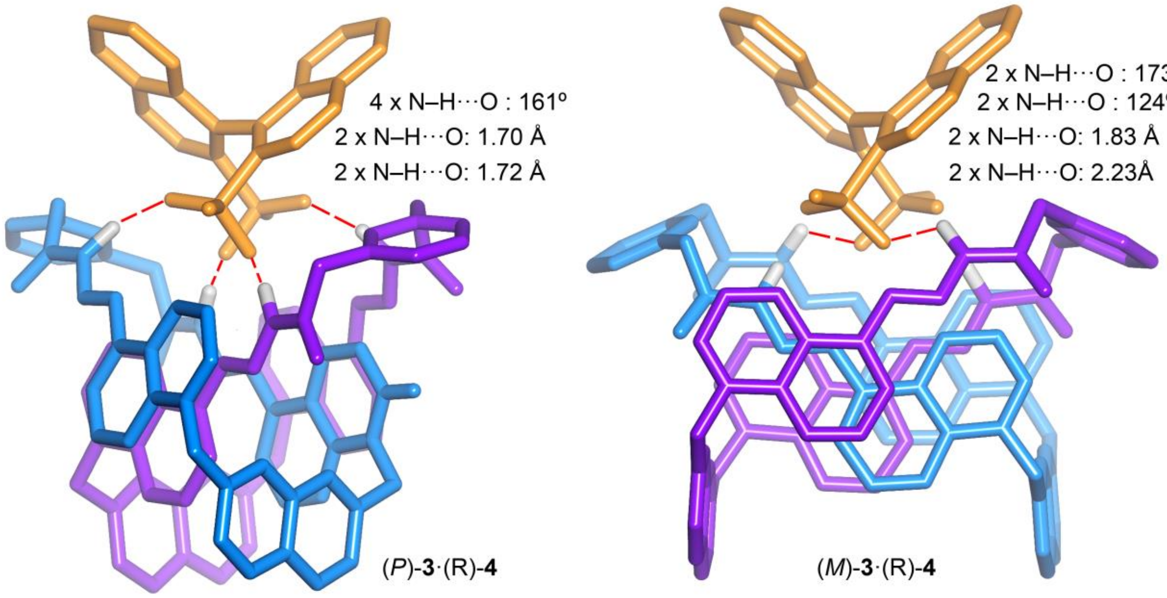

Figure S24. Optimized geometries of (*P*)-**3**·(*R*)-**4** and (*M*)-**3**·(*R*)-**4** complexes at the PB86-D3/def2-TZVP level of theory.

7.1 Cartesian coordinates of (*P*)-3·(*R*)-4

O	-5.46181745	-1.09665964	-0.77963915
O	-1.30449552	-5.58739138	1.12529595
N	-3.51303911	-0.29949004	0.15140641
H	-2.92836942	-0.18446894	1.02182471
N	-2.98434420	0.11437607	-1.01874271
N	1.88949341	3.26569174	-2.85936541
N	6.18320399	-2.52632283	-1.94990003
N	1.04517441	-4.26844641	0.79538607
N	0.32010295	-4.16577985	1.91461845
H	0.58213397	-3.44367857	2.62525743
C	-5.36969313	-4.95848361	2.28342598
H	-6.09941515	-5.76697032	2.21671657
C	-5.73356846	-3.65985719	1.92004417
H	-6.74825930	-3.45423535	1.57221225
C	-4.80658656	-2.61448126	2.01841487
C	-3.51082552	-2.89538984	2.46721646

H	-2.77563935	-2.09042690	2.54758748
C	-5.19663185	-1.19869440	1.63635730
H	-6.28837650	-1.09096922	1.66550613
H	-4.74840850	-0.46866972	2.32722506
C	-4.76805017	-0.87751855	0.21172461
C	-1.82808673	0.69895253	-0.90304438
H	-1.37520893	0.80659689	0.08802992
C	-1.13216663	1.22058842	-2.06227061
C	-1.61786842	1.01065382	-3.35185423
H	-2.53646598	0.44113122	-3.48068299
C	-0.94042842	1.51787166	-4.46502260
H	-1.33378563	1.32566622	-5.46333247
C	0.22498091	2.25666048	-4.32640989
H	0.73932101	2.61625991	-5.21117554
C	0.73559364	2.50932069	-3.03654271
C	0.08040432	1.97324916	-1.87648128
C	0.67427779	2.18717448	-0.60800798
H	0.25963301	1.74230700	0.29757182
C	1.82071987	2.95042074	-0.49401401
H	2.30096410	3.13142741	0.46276455
C	2.39987072	3.48945004	-1.63441680
H	3.30154942	4.09694033	-1.58604193
C	2.66947074	3.72963565	-4.03553713
H	3.35488533	4.50475711	-3.66800306
H	1.98925093	4.21757688	-4.74494434
C	3.42842569	2.58226448	-4.67168260
C	3.46055483	2.43406029	-6.08437406
H	2.93840655	3.15879649	-6.71446529
C	4.15164703	1.39179957	-6.66860986
H	4.17373711	1.29304369	-7.75444460
C	4.83943499	0.43832688	-5.87039805
C	5.54999376	-0.66681314	-6.41049015
H	5.59000838	-0.79837623	-7.49188612
C	6.18200270	-1.57262902	-5.58163054
H	6.71874755	-2.41890673	-6.01479740
C	6.14200061	-1.41276740	-4.17030397
C	5.47032428	-0.34201456	-3.61953168
H	5.41356108	-0.18204878	-2.54234463
C	4.81094399	0.59712692	-4.44570657
C	4.10065376	1.68003625	-3.87364817
H	4.09957126	1.75157055	-2.78352697
C	6.84706844	-2.41202422	-3.27385882
H	6.88847947	-3.40964257	-3.72854555
H	7.88381860	-2.10557442	-3.08029907
C	6.78343178	-1.97930717	-0.87956424
H	7.75873391	-1.52902483	-1.05300356
C	6.17841186	-1.97091709	0.37146202
H	6.70057506	-1.51432060	1.20843580
C	4.91047024	-2.50534680	0.51956419
H	4.42073617	-2.43838560	1.49306372
C	4.25415834	-3.09958169	-0.58257672
C	4.91935986	-3.10634976	-1.85816024
C	4.30832876	-3.67834622	-2.98604963
H	4.78702123	-3.65493947	-3.96016517
C	3.06010899	-4.27081310	-2.84847930
H	2.59389599	-4.72734919	-3.71975827
C	2.38874374	-4.28226632	-1.62480828
H	1.40659139	-4.74350393	-1.53418131
C	2.94611063	-3.69425222	-0.48510929
C	2.19674073	-3.66255686	0.76090564
H	2.59205304	-3.12561041	1.63193423
C	-0.90542729	-4.82766455	1.99286465
C	-1.70854111	-4.46829262	3.24463885
H	-1.65540258	-5.33132260	3.92569797
H	-1.27276446	-3.60528409	3.76788686
C	-3.13690948	-4.19308658	2.83917321
C	-4.07710305	-5.22489728	2.74117613
H	-3.79306610	-6.24117218	3.01604407
O	5.46145209	1.09699170	-0.77887764
O	1.30549343	5.58816757	1.12463775
N	3.51289438	0.30089385	0.15153872
H	2.92904207	0.18430504	1.02029160
N	2.98507705	-0.11346327	-1.01869454
N	-1.89010290	-3.26671015	-2.85980855
N	-6.18432517	2.52641231	-1.95006243
N	-1.04477237	4.26714886	0.79582963
N	-0.32175743	4.16484860	1.91478589

H	-0.58135160	3.44397228	2.62493961
C	5.36884356	4.95840719	2.28345552
H	6.09912181	5.76538971	2.21614728
C	5.73461110	3.66009017	1.92065777
H	6.74858836	3.45557704	1.57211436
C	4.80698996	2.61588642	2.01774404
C	3.50927507	2.89586202	2.46779307
H	2.77648040	2.09060958	2.54639773
C	5.19782147	1.19963582	1.63536415
H	6.28780910	1.09076420	1.66542788
H	4.74766178	0.47005886	2.32809633
C	4.76812750	0.87593287	0.21135528
C	1.82727846	-0.69958598	-0.90299228
H	1.37477922	-0.80728637	0.08823042
C	1.13261330	-1.21970064	-2.06293323
C	1.61709914	-1.01093213	-3.35293915
H	2.53664903	-0.44048881	-3.48159830
C	0.94141197	-1.51866130	-4.46546048
H	1.33412683	-1.32509058	-5.46383621
C	-0.22404208	-2.25670982	-4.32627651
H	-0.74012230	-2.61714635	-5.21129474
C	-0.73488125	-2.50826258	-3.03771140
C	-0.08105351	-1.97479938	-1.87651748
C	-0.67488472	-2.18749084	-0.60782329
H	-0.25943084	-1.74248072	0.29749284
C	-1.82209440	-2.95029285	-0.49402844
H	-2.30113736	-3.13210626	0.46280628
C	-2.39976736	-3.48890129	-1.63416593
H	-3.30115989	-4.09656507	-1.58588320
C	-2.66942981	-3.72961034	-4.03606353
H	-1.98856973	-4.21718286	-4.74372047
H	-3.35564518	-4.50518565	-3.66820607
C	-3.42872433	-2.58254359	-4.67156535
C	-3.46097867	-2.43425408	-6.08347340
H	-2.93902683	-3.15813110	-6.71422338
C	-4.15196819	-1.39220672	-6.66788232
H	-4.17498951	-1.29285051	-7.75445849
C	-4.83861375	-0.43810569	-5.86989604
C	-4.81002745	-0.59715115	-4.44530849
C	-4.10167822	-1.67876990	-3.87352383
H	-4.10080897	-1.75100683	-2.78226931
C	-5.47173993	0.34204998	-3.61959690
H	-5.41453280	0.18129564	-2.54226531
C	-6.14154314	1.41231726	-4.17168204
C	-6.18293562	1.57371590	-5.58235704
H	-6.72001971	2.41872859	-6.01569407
C	-5.54941440	0.66730430	-6.41025833
H	-5.58929399	0.79855240	-7.49272621
C	-6.84553081	2.41199737	-3.27374673
H	-7.88383916	2.10487642	-3.08074711
H	-6.88828779	3.40988115	-3.72964661
C	-6.78363518	1.97827897	-0.87844138
H	-7.75867115	1.52939430	-1.05289849
C	-6.17972729	1.97080739	0.37150738
H	-6.70078339	1.51385326	1.20779376
C	-4.91044445	2.50551743	0.51862277
H	-4.42171376	2.43742929	1.49395256
C	-4.25321282	3.09848463	-0.58196561
C	-4.91957086	3.10688848	-1.85762000
C	-4.30801113	3.67803786	-2.98677237
H	-4.78593869	3.65569269	-3.96025513
C	-3.06149313	4.27108568	-2.84760053
H	-2.59277907	4.72645960	-3.72036212
C	-2.38876106	4.28181957	-1.62445188
H	-1.40727204	4.74498088	-1.53326529
C	-2.94577251	3.69469617	-0.48485343
C	-2.19581352	3.66181557	0.76081354
H	-2.59231581	3.12586158	1.63283556
C	0.90466470	4.82857151	1.99249225
C	1.70855437	4.46941802	3.24384500
H	1.65656093	5.33187078	3.92650714
H	1.27163071	3.60568459	3.76691519
C	3.13811917	4.19186500	2.83952661
C	4.07704304	5.22599714	2.74034856
H	3.79227287	6.24053965	3.01637868
C	2.95283212	0.96534406	4.88444176
C	2.93483395	1.99915989	5.78571556

C	1.73464582	2.34389592	6.45822789
C	0.55805552	1.54700479	6.24257625
C	0.59406572	0.45515778	5.30676187
C	1.77587038	0.22525294	4.61213515
H	2.57987244	4.03669901	7.51056267
H	3.87371791	0.69359042	4.37049774
H	3.84207522	2.57104182	5.98374039
C	1.67764163	3.44140459	7.35512385
C	-0.61927736	1.87746678	6.96804697
C	-0.64169904	2.95307949	7.83259460
C	0.51306340	3.74600404	8.02754295
H	-1.51753146	1.28127662	6.82653228
H	-1.55728007	3.19316196	8.37218517
H	0.48255208	4.58930012	8.71801225
C	-0.59461530	-0.45500098	5.30687748
C	-0.55775892	-1.54699523	6.24185127
C	-1.77650590	-0.22455163	4.61284464
C	-1.73393700	-2.34312843	6.45819664
C	0.61845983	-1.87836018	6.96856603
C	-2.95386619	-0.96534169	4.88385855
C	-1.67815569	-3.44075571	7.35647271
C	-2.93363304	-1.99918573	5.78453043
C	0.64100804	-2.95332572	7.83256078
H	1.51902516	-1.28135652	6.82716841
H	-3.87453648	-0.69325106	4.37095893
C	-0.51314488	-3.74532543	8.02762694
H	-2.57941079	-4.03729075	7.51036982
H	-3.84223734	-2.57227013	5.98423013
H	1.55764876	-3.19213643	8.37165225
H	-0.48206830	-4.59061251	8.71780028
S	-1.87274233	0.87887921	3.20869734
S	1.87342362	-0.87917201	3.20839728
O	-1.57659665	-0.01070624	2.03566671
O	-3.25882068	1.39139683	3.12353713
O	-0.84878647	1.92652072	3.38671484
O	0.84962780	-1.92671458	3.38613225
O	3.25882537	-1.39096544	3.12231698
O	1.57514372	0.01044078	2.03692209

7.2 Cartesian coordinates of (*M*)-3·(*R*)-4

O	-5.5190658	-0.2158161	0.2974342
O	-1.9912069	-5.8892994	-0.6925013
N	-3.3637544	0.0055180	-0.4781181
H	-2.7526951	-0.0500481	-1.3274935
N	-2.8734127	0.4512795	0.6970915
N	2.1204943	3.1730731	2.8415897
N	5.8523279	-3.0259617	1.7430710
N	0.4628646	-4.7131528	-0.5076302
N	-0.2967328	-4.6444827	-1.6147430
H	-0.0287954	-3.9808592	-2.3551094
C	-5.8543702	-4.4278327	-1.4640727
H	-6.6608264	-5.0323691	-1.0470958
C	-5.9783018	-3.0385077	-1.5126725
H	-6.8698314	-2.5512550	-1.1152866
C	-4.9567589	-2.2583971	-2.0700215
C	-3.8051519	-2.8831527	-2.5709240
H	-3.0037108	-2.2727552	-2.9974196
C	-5.0615797	-0.7452282	-2.0323319
H	-6.0934654	-0.4251323	-2.2255311
H	-4.3931546	-0.2834772	-2.7721226
C	-4.7079701	-0.2875808	-0.6236151
C	-1.6247673	0.8107319	0.6739733
H	-1.0591199	0.7809952	-0.2634051
C	-0.9749480	1.3163746	1.8730883
C	-1.5351258	1.1259451	3.1338248
H	-2.4869260	0.6042524	3.2136712
C	-0.8798883	1.5707038	4.2876532
H	-1.3298945	1.3811100	5.2623431
C	0.3387780	2.2292094	4.2200567
H	0.8379868	2.5299550	5.1370893
C	0.9193421	2.4764101	2.9600430

C	0.2857139	2.0051799	1.7633255
C	0.9387987	2.2152334	0.5261720
H	0.4972367	1.8680683	-0.4095938
C	2.1419548	2.8927560	0.4735329
H	2.6673947	3.0566270	-0.4643179
C	2.6978206	3.3832248	1.6432331
H	3.6301750	3.9442976	1.6457799
C	2.8971063	3.5369454	4.0487117
H	3.6064427	4.3175706	3.7427083
H	2.2269967	3.9934770	4.7872186
C	3.6212484	2.3280056	4.6100020
C	3.7885458	2.1962247	6.0130617
H	3.3751519	2.9566222	6.6789647
C	4.4778635	1.1236478	6.5419730
H	4.6067846	1.0362840	7.6220287
C	5.0282134	0.1242551	5.6962541
C	5.7406671	-1.0021732	6.1875807
H	5.8868385	-1.1128548	7.2632310
C	6.2421343	-1.9500150	5.3197164
H	6.7848746	-2.8115032	5.7146232
C	6.0601506	-1.8166571	3.9174141
C	5.3797337	-0.7300741	3.4076328
H	5.2254094	-0.5992142	2.3329015
C	4.8545217	0.2558624	4.2788699
C	4.1483175	1.3705786	3.7647611
H	4.0327139	1.4290085	2.6801008
C	6.6337690	-2.8718696	2.9941853
H	6.6859043	-3.8518416	3.4884625
H	7.6578492	-2.6123961	2.6938418
C	6.3974159	-2.6129896	0.5872802
H	7.3980236	-2.1918049	0.6517192
C	5.7075777	-2.6963987	-0.6168154
H	6.1858756	-2.3391074	-1.5256306
C	4.4121562	-3.1771115	-0.6278099
H	3.8594729	-3.1646202	-1.5693822
C	3.8075915	-3.6238396	0.5708634
C	4.5588680	-3.5446705	1.7927037
C	4.0014131	-3.9765275	3.0087463
H	4.5505471	-3.8950789	3.9422401
C	2.7152460	-4.4972025	3.0143780
H	2.2870857	-4.8379502	3.9573451
C	1.9579600	-4.5835531	1.8435643
H	0.9465053	-4.9879497	1.8637615
C	2.4709369	-4.1548045	0.6177738
C	1.6576141	-4.2150764	-0.5862645
H	2.0461578	-3.8064600	-1.5235589
C	-1.5616715	-5.2313918	-1.6255883
C	-2.3654473	-4.9370698	-2.8953578
H	-2.5510084	-5.9057553	-3.3833253
H	-1.8009372	-4.3070283	-3.5960903
C	-3.6671722	-4.2774081	-2.5014630
C	-4.7006670	-5.0456342	-1.9480239
H	-4.5883196	-6.1277441	-1.8840354
O	5.5190658	0.2158161	0.2974342
O	1.9912069	5.8892994	-0.6925013
N	3.3637544	-0.0055180	-0.4781181
H	2.7526951	0.0500481	-1.3274935
N	2.8734127	-0.4512795	0.6970915
N	-2.1204943	-3.1730731	2.8415897
N	-5.8523279	3.0259617	1.7430710
N	-0.4628646	4.7131528	-0.5076302
N	0.2967328	4.6444827	-1.6147430
H	0.0287954	3.9808592	-2.3551094
C	5.8543702	4.4278327	-1.4640727
H	6.6608264	5.0323691	-1.0470958
C	5.9783018	3.0385077	-1.5126725
H	6.8698314	2.5512550	-1.1152866
C	4.9567589	2.2583971	-2.0700215
C	3.8051519	2.8831527	-2.5709240
H	3.0037108	2.2727552	-2.9974196
C	5.0615797	0.7452282	-2.0323319
H	6.0934654	0.4251323	-2.2255311
H	4.3931546	0.2834772	-2.7721226
C	4.7079701	0.2875808	-0.6236151
C	1.6247673	-0.8107319	0.6739733
H	1.0591199	-0.7809952	-0.2634051
C	0.9749480	-1.3163746	1.8730883

C	1.5351258	-1.1259451	3.1338248
H	2.4869260	-0.6042524	3.2136712
C	0.8798883	-1.5707038	4.2876532
H	1.3298945	-1.3811100	5.2623431
C	-0.3387780	-2.2292094	4.2200567
H	-0.8379868	-2.5299550	5.1370893
C	-0.9193421	-2.4764101	2.9600430
C	-0.2857139	-2.0051799	1.7633255
C	-0.9387987	-2.2152334	0.5261720
H	-0.4972367	-1.8680683	-0.4095938
C	-2.1419548	-2.8927560	0.4735329
H	-2.6673947	-3.0566270	-0.4643179
C	-2.6978206	-3.3832248	1.6432331
H	-3.6301750	-3.9442976	1.6457799
C	-2.8971063	-3.5369454	4.0487117
H	-2.2269967	-3.9934770	4.7872186
H	-3.6064427	-4.3175706	3.7427083
C	-3.6212484	-2.3280056	4.6100020
C	-3.7885458	-2.1962247	6.0130617
H	-3.3751519	-2.9566222	6.6789647
C	-4.4778635	-1.1236478	6.5419730
H	-4.6067846	-1.0362840	7.6220287
C	-5.0282134	-0.1242551	5.6962541
C	-4.8545217	-0.2558624	4.2788699
C	-4.1483175	-1.3705786	3.7647611
H	-4.0327139	-1.4290085	2.6801008
C	-5.3797337	0.7300741	3.4076328
H	-5.2254094	0.5992142	2.3329015
C	-6.0601506	1.8166571	3.9174141
C	-6.2421343	1.9500150	5.3197164
H	-6.7848746	2.8115032	5.7146232
C	-5.7406671	1.0021732	6.1875807
H	-5.8868385	1.1128548	7.2632310
C	-6.6337690	2.8718696	2.9941853
H	-7.6578492	2.6123961	2.6938418
H	-6.6859043	3.8518416	3.4884625
C	-6.3974159	2.6129896	0.5872802
H	-7.3980236	2.1918049	0.6517192
C	-5.7075777	2.6963987	-0.6168154
H	-6.1858756	2.3391074	-1.5256306
C	-4.4121562	3.1771115	-0.6278099
H	-3.8594729	3.1646202	-1.5693822
C	-3.8075915	3.6238396	0.5708634
C	-4.5588680	3.5446705	1.7927037
C	-4.0014131	3.9765275	3.0087463
H	-4.5505471	3.8950789	3.9422401
C	-2.7152460	4.4972025	3.0143780
H	-2.2870857	4.8379502	3.9573451
C	-1.9579600	4.5835531	1.8435643
H	-0.9465053	4.9879497	1.8637615
C	-2.4709369	4.1548045	0.6177738
C	-1.6576141	4.2150764	-0.5862645
H	-2.0461578	3.8064600	-1.5235589
C	1.5616715	5.2313918	-1.6255883
C	2.3654473	4.9370698	-2.8953578
H	2.5510084	5.9057553	-3.3833253
H	1.8009372	4.3070283	-3.5960903
C	3.6671722	4.2774081	-2.5014630
C	4.7006670	5.0456342	-1.9480239
H	4.5883196	6.1277441	-1.8840354
C	0.4142346	-3.0790315	-4.8169427
C	-0.5139566	-3.5008637	-5.7357798
C	-1.3558650	-2.5634801	-6.3893567
C	-1.1621497	-1.1609785	-6.1427298
C	-0.1583027	-0.7306966	-5.2032438
C	0.5643072	-1.6969007	-4.5246213
H	-2.4917433	-4.0490825	-7.4814354
H	1.0798360	-3.7928464	-4.3289320
H	-0.6093201	-4.5610302	-5.9775506
C	-2.3583678	-2.9805590	-7.3020055
C	-1.9806032	-0.2384421	-6.8487077
C	-2.9515243	-0.6766141	-7.7265896
C	-3.1464370	-2.0572626	-7.9565644
H	-1.8454083	0.8296803	-6.6865724
H	-3.5717902	0.0504488	-8.2518842
H	-3.9135087	-2.3885198	-8.6571721
C	0.1583027	0.7306966	-5.2032438

C	1.1621497	1.1609785	-6.1427298
C	-0.5643072	1.6969007	-4.5246213
C	1.3558650	2.5634801	-6.3893567
C	1.9806032	0.2384421	-6.8487077
C	-0.4142346	3.0790315	-4.8169427
C	2.3583678	2.9805590	-7.3020055
C	0.5139566	3.5008637	-5.7357798
C	2.9515243	0.6766141	-7.7265896
H	1.8454083	-0.8296803	-6.6865724
H	-1.0798360	3.7928464	-4.3289320
C	3.1464370	2.0572626	-7.9565644
H	2.4917433	4.0490825	-7.4814354
H	0.6093201	4.5610302	-5.9775506
H	3.5717902	-0.0504488	-8.2518842
H	3.9135087	2.3885198	-8.6571721
S	-1.5633075	1.3898424	-3.0721421
S	1.5633075	-1.3898424	-3.0721421
O	-0.6540653	1.8881492	-1.9982956
O	-2.7804562	2.2213853	-3.1780558
O	-1.8511235	-0.0552601	-2.9216593
O	1.8511235	0.0552601	-2.9216593
O	2.7804562	-2.2213853	-3.1780558
O	0.6540653	-1.8881492	-1.9982956

8. References

S1. http://mestrelab.com/software/freeware/
S2. Sheldrick, G. *Acta Crystallographica Section C* **2015**, *71*, 3-8.
S3. Dolomanov, O. V.; Bourhis, L. J.; Gildea, R. J.; Howard, J. A. K.; Puschmann, H. *J. Appl. Crystallogr.* **2009**, *42*, 339-341.
S4. Zahmatkesh, S. *Lett. Org. Chem.* **2015**, *12*, 663-667.
S5. Ahlrichs, R.; Bär, M.; Hacer, M.; Horn, H.; Kömel, C. *Chem. Phys. Lett.* **1989**, *162*, 165–169.
S6. Grimme, S.; Antony, J.; Ehrlich, S.; Krieg, H. *J. Chem. Phys.* **2010**, *132*, 154104.
S7. Klamt, A.; Schüürmann, G. *J. Chem. Soc., Perkin Trans. 2* **1993**, 799-805.
S8. Klamt, A. *WIREs Comput. Mol. Sci.* **2011**, *1*, 699-709.
